# Cohort event monitoring of safety of COVID-19 vaccines: the Italian experience of the “ilmiovaccinoCOVID19 collaborating group”

**DOI:** 10.3389/fdsfr.2024.1363086

**Published:** 2024-08-12

**Authors:** Nicoletta Luxi, Chiara Bellitto, Francesco Ciccimarra, Emiliano Cappello, Luca L’Abbate, Marco Bonaso, Chiara Ajolfi, Paolo Baldo, Roberto Bonaiuti, Claudio Costantino, Giovambattista De Sarro, Cristina Di Mauro, Giuseppina Fava, Marina Ferri, Alberto Firenze, Fabiana Furci, Luca Gallelli, Luca Leonardi, Giovanna Negri, Fabio Pieraccini, Elisabetta Poluzzi, Chiara Sacripanti, Elisa Sangiorgi, Ester Sapigni, Ilenia Senesi, Roberto Tessari, Luigia Trabace, Alfredo Vannacci, Francesca Venturini, Francesco Vitale, Donatella Zodda, Marco Tuccori, Gianluca Trifirò

**Affiliations:** ^1^ Department of Medicine, University of Verona, Verona, Italy; ^2^ Department of Diagnostics and Public Health, University of Verona, Verona, Italy; ^3^ Unit of Pharmacology and Pharmacovigilance, Department of Clinical and Experimental Medicine, University of Pisa, Pisa, Italy; ^4^ Modena Local Health Unit, Modena, Italy; ^5^ Hospital Pharmacy Unit, Centro di Riferimento Oncologico di Aviano, National Cancer Institute, IRCCS, Aviano, Italy; ^6^ PeaRL-Perinatal Research Laboratory, Department of Neurosciences, Psychology, Drug Research and Child Health, CiaoLapo Foundation, University of Florence, Florence, Italy; ^7^ Department of Health Promotion, Maternal and Infant Care, Internal Medicine and Excellence Specialties “G. D’Alessandro” - University of Palermo, Palermo, Italy; ^8^ Department of Health Sciences, School of Medicine and Surgery, Magna Graecia University of Catanzaro, Catanzaro, Italy; ^9^ Caserta Local Health Unit, Caserta, Italy; ^10^ Reggio Calabria Local Health Unit, Reggio Calabria, Italy; ^11^ Autonomous Province of Trento, Pharmacovigilance Centre, Trento, Italy; ^12^ Struttura Commissariale dell’Azienda Ospedaliera Papardo, Messina, Italy; ^13^ Provincial Healthcare Unit, Section of Allergy, Vibo Valentia, Italy; ^14^ Parma Local Health Unit, Parma, Italy; ^15^ Romagna Local Health Unit, Romagna, Italy; ^16^ Pharmacology Unit, Department of Medical and Surgical Sciences, University of Bologna, Bologna, Italy; ^17^ Bologna Local Health Unit, Bologna, Italy; ^18^ Area Governo del Farmaco e dei Dispositivi Medici, Regione Emilia Romagna, Bologna, Italy; ^19^ Emilia-Romagna Pharmacovigilance Regional Centre, Bologna, Italy; ^20^ Abruzzo Region, Pharmacovigilance Regional Centre, Bologna, Italy; ^21^ IRCCS Ospedale Sacro Cuore Don Calabria—Hospital Pharmacy, Negrar di Valpolicella, Italy; ^22^ Department of Clinical and Experimental Medicine, University of Foggia, Foggia, Italy; ^23^ Pharmacy Department, University Hospital of Padua, Padua, Italy; ^24^ Messina Local Health Unit, Messina, Italy

**Keywords:** active surveillance, vaccine safety, COVID-19, special cohorts, passive surveillance

## Abstract

**Introduction:** In 2021, the European Medicines Agency supported the “Covid Vaccine Monitor (CVM),” an active surveillance project spanning 13 European countries aimed at monitoring the safety of COVID-19 vaccines in general and special populations (i.e., pregnant/breastfeeding women, children/adolescents, immunocompromised people, and people with a history of allergies or previous SARS-CoV-2 infection). Italy participated in this project as a large multidisciplinary network called the “ilmiovaccinoCOVID19 collaborating group.”

**Methods:** The study aimed to describe the experience of the Italian network “ilmiovaccinoCOVID19 collaborating group” in the CVM context from June 2021 to February 2023. Comprising about 30 partners, the network aimed to facilitate vaccinee recruitment. Participants completed baseline and follow-up questionnaires within 48 h from vaccination over a 6-month period. Analyses focused on those who completed both the baseline and the first follow-up questionnaire (Q1), exploring temporal trends, vaccination campaign correlation, and loss to follow-up. Characteristics of recruited vaccinees and vaccinee-reported adverse drug reactions (ADRs) were compared with passive surveillance data in Italy.

**Results:** From June 2021 to November 2022, 22,384,663 first doses and 38,207,452 booster doses of COVID-19 vaccines were administered in Italy. Simultaneously, the study enrolled 1,229 and 2,707 participants for the first and booster doses, respectively. Of these, 829 and 1,879 vaccinees, respectively, completed both baseline and at least Q1 and were included in the analyses, with a significant proportion of them (57.8%/34.3%) belonging to special cohorts. Most vaccinees included in the analyses were women. Comirnaty^®^ (69%) and Spikevax^®^ (29%) were the most frequently administered vaccines. ADR rates following Comirnaty^®^ and Spikevax^®^ were higher after the second dose, particularly following Spikevax^®^. Serious ADRs were infrequent. Differences were observed in ADR characteristics between CVM and Italian passive surveillance.

**Conclusion:** This study confirmed the favorable safety profile of COVID-19 vaccines, with findings consistent with pivotal clinical trials of COVID-19 vaccines, although different proportions of serious ADRs compared to spontaneous reporting were observed. Continuous evaluation through cohort event monitoring studies provides real-time insights crucial for regulatory responses. Strengthening infrastructure and implementing early monitoring strategies are essential to enhance vaccine safety assessment and prepare for future pandemics.

## 1 Introduction

The rapid spread of coronavirus disease 2019 (COVID-19) worldwide due to severe acute respiratory syndrome coronavirus 2 (SARS-CoV-2) triggered the need to rapidly develop vaccines in response to this pandemic. The European Medicines Agency (EMA) conditionally approved Comirnaty, the first marketed COVID-19 vaccine, on 21 December 2020. Eight COVID-19 vaccines are currently available in the European Union (European Medicines Agency, 2023a). The unprecedented pace at which COVID-19 vaccines have been approved led to increased uncertainty about their efficacy and safety (Rosenthal et al., 2021). Although the benefit–risk profile of these vaccines was proven to be favorable in pre-authorization clinical trials, at the time of marketing, long-term effects were not adequately investigated, and despite several thousands of persons being recruited into pivotal trials, rare and serious adverse reactions (ADRs) could not be excluded (Doshi, 2020; Janiaud et al., 2021; World Health Organization, 2022). In addition, vulnerable populations (e.g., children and adolescents, pregnant and lactating women, people with allergies, and immune-compromised) were not initially included in pivotal clinical trials. As such, thorough re-evaluation of benefit–risk profiles of COVID-19 vaccines through passive and active surveillance in the post-marketing setting was of paramount importance. Accordingly, several large-scale real-world studies have been funded by international regulatory agencies (European Medicines Agency, 2023b). In general, spontaneous reporting of suspected ADRs remains the cornerstone for post-marketing vaccine safety surveillance and specifically for signal detection using different measures of disproportionality, such as the reporting odds ratio and observed *versus* expected analysis (Bate et al., 2009). The latter relies on the availability of information on background incidence rates, especially for adverse events of special interest (AESIs) from different data sources. With respect to that, the Coalition for Epidemic Preparedness Innovations (CEPI), together with the Brighton Collaboration, has created a preliminary list of AESIs for COVID-19 vaccine safety monitoring (Brighton Collaboration, 2020). Thereafter, the EMA-funded vACCine COVID-19 monitoring readinESS (ACCESS) project generated background incidence rates of 41 AESIs to contextualize potential safety signals detected following the administration of COVID-19 vaccines (Willame et al., 2023).

To integrate evidence from the passive surveillance, cohort event monitoring (CEM) provides a more comprehensive overview of COVID-19 vaccine safety, especially in those categories of vaccinees who are usually excluded from pivotal trials. Several active surveillance studies have been implemented worldwide to provide further insights on the post-marketing safety of COVID-19 vaccines in a rapid context. In particular, V-safe was a smartphone-based national surveillance system for COVID-19 vaccine safety implemented in the United States that allowed newly vaccinated people to report post-vaccination safety-related information (Meyers et al., 2023). Similarly, the COVID Symptom Study was launched in the United Kingdom (Menni et al., 2021). In Australia, vaccinees recruited at the community pharmacies participating in active vaccine safety surveillance could report COVID-19 vaccine-related suspected ADRs through the SmartVax tool (Salter et al., 2022). In Israel, the PerMed study was conducted to evaluate the safety profile of the second booster COVID-19 vaccine (Yechezkel et al., 2023). In Europe, the “Covid Vaccine Monitor” (CVM) project, an active surveillance program involving multiple countries, was started in February 2021 (EU PAS number 42504). This project was aimed at monitoring the safety of COVID-19 vaccines in the general population as well as in a special cohort of vulnerable patients (i.e., pregnant and lactating woman, children and adolescents, immunocompromised, people with history of allergy, and people with prior SARS-CoV-2 infection), collecting vaccinee-reported ADRs via dedicated web apps in 13 European countries, including Italy, which participated to the project as a large multidisciplinary network “ilmiovaccinoCOVID19 collaborating group.”

The purpose of this study was to describe the main findings and lessons learned from the “ilmiovaccinoCOVID19 collaborating group,” the Italian research network, which contributed to recruiting COVID-19 vaccinees as part of the CVM project. In detail, we reported findings from the active surveillance of the first vaccination cycle and the booster dose of any EMA-authorized COVID-19 vaccine, spanning from June 2021 to February 2023 in Italy, highlighting the main methodological challenges that have been encountered. The study encompassed vaccinees from the general population as well as special cohorts of vulnerable vaccinees, using electronic questionnaires for data collection. We have also compared the information collected in this active surveillance study to those reported in passive surveillance based on a spontaneous reporting system in Italy.

## 2 Methods

### 2.1 The Italian network “ilmiovaccinoCOVID19 collaborating group”

**FIGURE 1:**
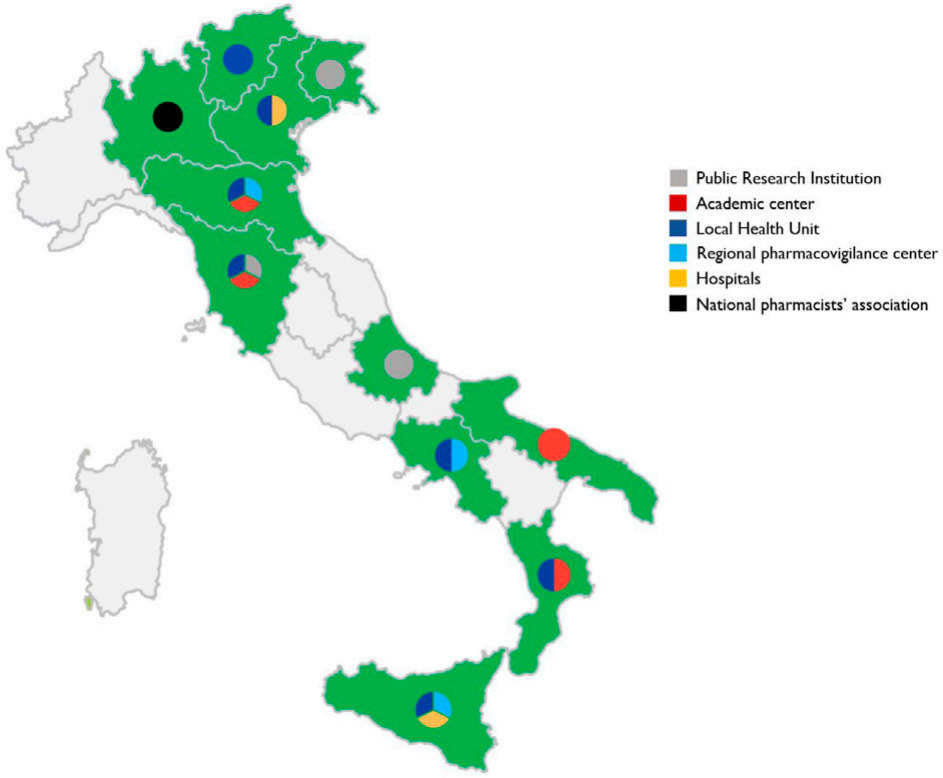
Distribution of partners participating in the “ilmiovaccinocovid19 collaborating group” throughout Italy.

Italy participated in the CVM project through a large network named ‘ilmiovaccinocovid19 collaborating group’, coordinated by the University of Verona. The Italian network involved about 30 partners all over the country (Figure 1), including regional pharmacovigilance centers, academic centers, national scientific societies, patient organizations, as well as public hospitals and local health units covering overall around 100 dedicated COVID-19 vaccination centers. The network was set up with the aim of facilitating the dissemination of the study and the recruitment of vaccinees by supporting them in the web-based registration as well as in completing the baseline questionnaires on a voluntary basis.

Dissemination material, including flyers, posters, animation videos, and infographics (Supplementary Material S1), was distributed nationwide through channels such as print magazines, online journals, scientific society web pages, social networks, and information desks in vaccination centers.

### 2.2 Setting and study population

This prospective cohort study was carried out using web-based questionnaires collecting information on vaccinee’s characteristics at baseline and vaccinee-reported ADRs in the follow-up questionnaires from 9 June 2021 to 28 February 2023 in Italy. All vaccinees who registered in the web app within 48 h after receiving either a first dose or a booster dose of any EMA-authorized COVID-19 vaccine and provided an electronic informed consent were enrolled in the study from 9 June 2021 to 30 November 2022. Specifically, people receiving the first dose of the vaccine were enrolled from 9 June 2021 to 31 August 2022; people who received the booster dose of the vaccine were enrolled from 27 October 2021 to 30 November 2022. In addition, active recruitment was specifically sought for the following vulnerable populations: pregnant and lactating women, children and adolescents aged between 5 and 17 years, immunocompromised patients, and people with a history of allergy or with prior SARS-CoV-2 infection. Pregnant women at any point of pregnancy at the time of vaccination or during the breastfeeding period were included in the special cohort. Immunocompromised subjects were defined as subjects with immune system compromised due to a disease (e.g., HIV/AIDS, transplants, autoimmune diseases, leukemia/lymphoma) and/or subjects under treatment affecting their immune system (e.g., myelosuppressive chemotherapy, glucocorticoids, anti-rheumatics drugs, or monoclonal antibodies interfering with the immune system). Subjects with a history of allergy, including hay fever, dust mite allergy, allergy to animals, food allergy, allergy to insect bites, allergy to medication or vaccine, etc., were included in the special cohort. People with prior SARS-CoV-2 infection were defined as people who had a suspected/diagnosed SARS-CoV-2 infection (whether confirmed or not-confirmed by a test) at any time prior to the first dose vaccination. For children and adolescent vaccinees (<18 years old), parents or legal representatives were able to participate in the study on their behalf. Vaccinees could belong to more than one special cohort.

The study protocol was approved by the Ethical Committee of the Spallanzani Hospital (Rome), the unique national committee for the investigation of COVID-19, with protocol number 463, and a Data Privacy Impact Assessment (DPIA) was signed.

To describe the differences from passive surveillance, we also used information published in periodic pharmacovigilance public reports available on the Italian Medicines Agency (2023) website. These reports contain information about the number of ADRs received during the observation period, the number of vaccines administered (overall and stratified by specific vaccine), reporting rate, and ADR distribution by seriousness and system organ class (SOC) codified using the MedDRA dictionary. Given the extremely dynamic scenario of the COVID-19 vaccination campaign, the number and the characteristics of reported suspected adverse reactions changed over time. In addition, published reports did not allow for retrieving information for a period that perfectly overlapped with the period of the study. For these reasons, we decided to extract information using the report covering the first quarter of the vaccination campaign, the first 6 months, and the final report, covering approximately 2 years of observation (AIFA, 2023b; AIFA 2023c; AIFA 2023a).

### 2.3 Data collection

Two different web-based apps with similar structure were developed for data collection: the Lareb-managed Intensive Monitoring (LIM) (developed by the Netherlands pharmacovigilance center Lareb) and the ResearchOnline (RO) (developed by the University Medical Center Utrecht). Both were built specifically for vaccinee-reported outcomes. In particular, the LIM app was designed to collect information on the safety of the first vaccination cycle only and for a limited time period, while the RO app also collected information on the safety of the booster dose.

**FIGURE 2:**
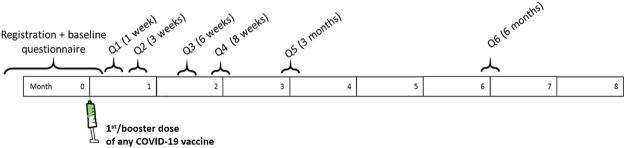
Questionnaire scheduling schemes over time for vaccinees recruited at the first or booster dose.

In detail, participants, after registering and providing informed consent, were invited via e-mail to complete the baseline questionnaire. Vaccinees who were not able to participate themselves (e.g., under-18 and older people) could participate via a proxy (e.g., a family member). In the baseline questionnaire (Annex I), information on the vaccinee’s characteristics, such as age and gender, comorbidities, prior SARS-CoV-2 infection, and concomitant drug use, as well as data on COVID-19 vaccine exposure (i.e., vaccine brand, batch, and dose, date of administration), was collected. Afterward, six follow-up questionnaires at different time points over 6 months from the vaccine administration date were sent to the participants to collect information on vaccinee-reported short and medium-term ADRs (Annex I); for vaccinees who were recruited at the booster dose, five follow-up questionnaires were sent over a 3-month period (Figure 2). If vaccinees reported any ADR in a specific follow-up questionnaire, questions regarding the outcome of the ADR were asked in the subsequent follow-up questionnaire.

For pregnant women, the schedule of follow-up questionnaires was planned based on the gestational period at the time of study enrollment; in addition, they were followed up until 1.5 months after the end of pregnancy to collect information on pregnancy and newborn outcomes.

In general, follow-up questionnaires collected information on a set of pre-specified (solicited) ADRs, both local (injection site hematoma, induration, inflammation, pain, pruritus, swelling, and warmth) and systemic ADRs (arthralgia, chills, fatigue, headache, malaise, myalgia, nausea, and fever) as well as unsolicited (reported as free text), with special attention to AESIs and serious ones. AESIs were defined based on the list established by the ACCESS project (Willame et al., 2023). The coding of ADRs reported in the questionnaires, both solicited and unsolicited ADRs reported as free text, was performed according to the MedDRA dictionary, version 24.0 (MedDRA, 2021). Solicited ADRs were automatically coded, while unsolicited ADRs were manually assessed and coded. This process, along with the assessment of the ADRs, was carried out by trained pharmacovigilance personnel. The seriousness of ADRs was assessed based on the Council for International Organizations of Medical Sciences (CIOMS) criteria (CIOMS, 2010). When first received, the information in the questionnaires might be incomplete or require more accurate information. If further investigation was needed, in some cases participants could be contacted via e-mail to provide additional information about the reported reactions. The data processing has already been described elsewhere (Raethke et al., 2023).

### 2.4 Data analysis

The aggregated data collected through the two tools were analyzed using a common data model (CDM) approach. The CDM enabled data harmonization and in-depth analysis at the individual vaccinee record level, as described elsewhere (Luxi et al., 2023; Raethke et al., 2023).

Time to vaccinee recruitment over the study period in relation to the number of cumulative administered first and booster COVID-19 vaccine doses in Italy has been reported. The time to vaccinee recruitment was defined as the date of the baseline questionnaire fulfillment. COVID-19 vaccine administration data in Italy were obtained from the surveillance bulletin provided by the Civil Protection Department (Civil Protection Department, 2023). We reported the number of questionnaires completed, including baseline and follow-up questionnaires, for vaccinees recruited at both the first vaccination cycle and the booster dose and belonging to different cohorts. Among vaccinees who registered for the study, only those who completed the baseline questionnaire plus at least the first follow-up questionnaire (i.e., Q1) were retained for the analyses. Descriptive statistics were used to describe the baseline characteristics of those vaccinees, stratified by a special cohort of vulnerable vaccinees. Children in the age category 0–4 years were included to give a complete overview of recruited vaccinees, even though they were enrolled before the approval of vaccination in children aged between 6 months and 4 years, but they were excluded from the analyses. To better explore if any selective loss to follow-up occurred, we compared the characteristics of vaccinees who completed only the baseline questionnaires vs. those who completed the baseline plus at least Q1. To establish whether there were statistically significant differences between the two groups of vaccinees, the proportions were compared using the χ^2^ test or Fisher’s exact test. Only *p*-values of 0.05 were considered statistically significant. In addition, for each dose, the rate of ADR was calculated as the proportion of vaccinees who reported ADRs after dose 1, 2, or booster out of the total number of recruited vaccinees who completed baseline plus at least Q1 at dose 1, 2, or booster. Specifically, the rate of any ADR, as a whole and specifically for solicited/unsolicited and serious ADRs, was calculated. Moreover, a list including all the reported ADRs by system organ class (SOC) was also provided.

Data from this study (active surveillance) and passive surveillance have been compared by calculating distributions of ADR reports (as percentages of the total) by seriousness, medicinal product, and SOC. Overall, reporting rates using the number of administered doses as a denominator have also been reported.

## 3 Results

**FIGURE 3:**
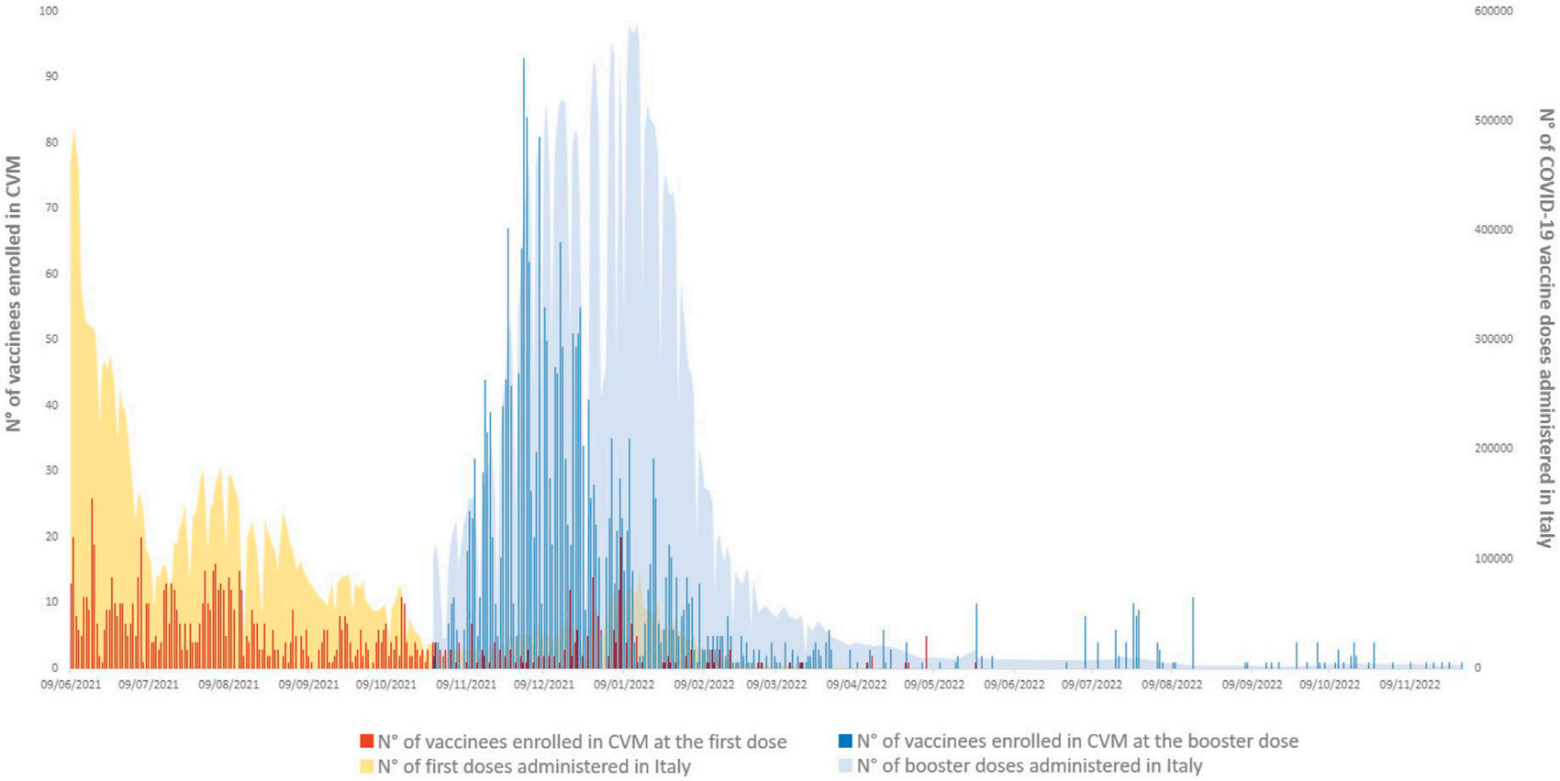
Cumulative number of recruited vaccinees at first or booster vaccine doses vs. the number of total cumulative administered first or booster vaccine doses in Italy during the recruitment period 24 June 2021–24 November 2022.

Overall, 22,384,663 first doses and 38,207,452 booster doses of COVID-19 vaccines were administered in Italy between June 2021 and November 2022. During the same period, 1,229 and 2,707 vaccinees receiving first and booster doses, respectively, were enrolled in the study (Figure 3).

**FIGURE 4:**
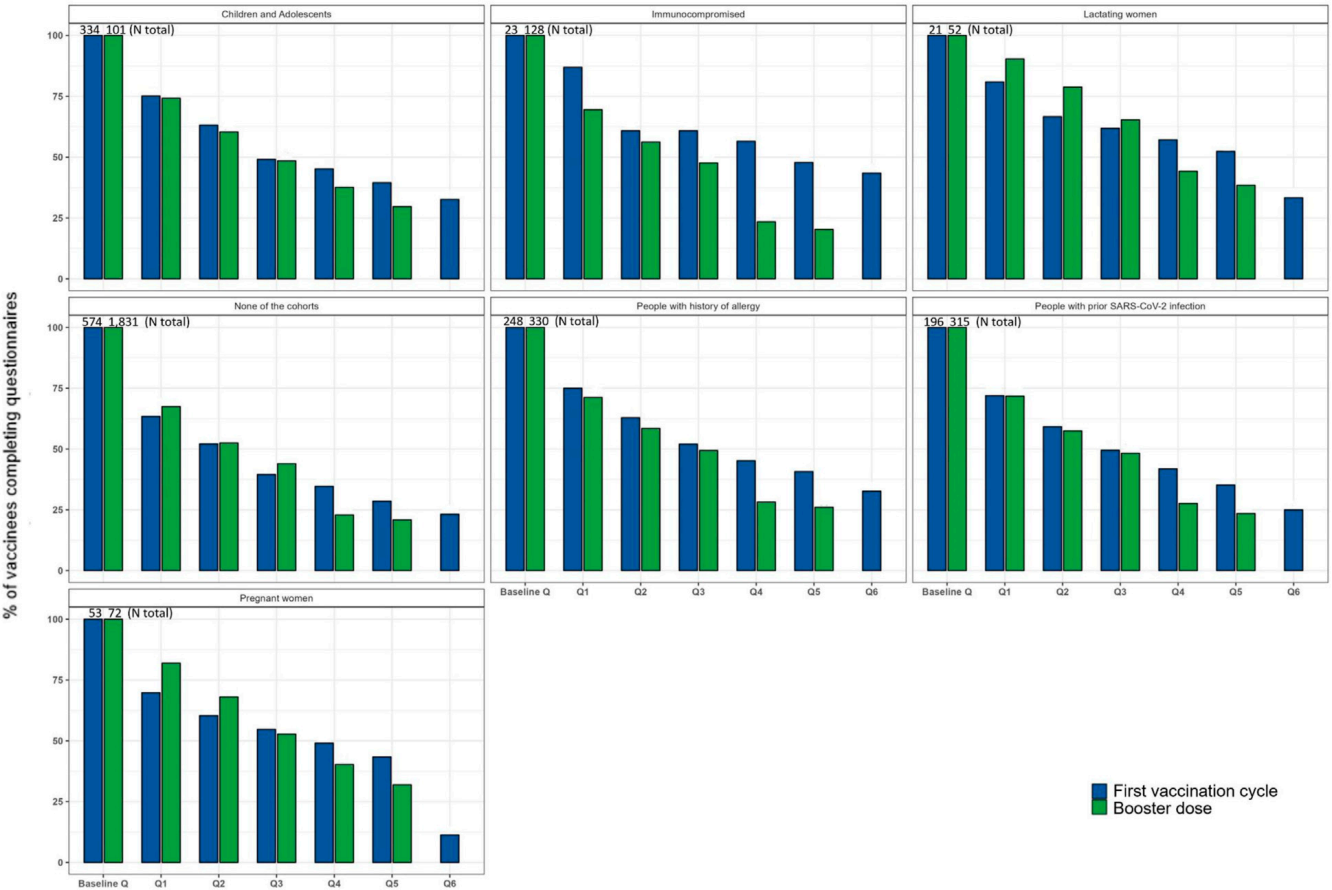
Frequency of vaccinees who completed the baseline and follow-up questionnaires by a special cohort and dose.

Among vaccinees who registered for the study, only those who completed the baseline questionnaire and at least the Q1 were included in the analyses. More than 70% of the vaccinees enrolled in the different cohorts at both the first and the booster dose completed at least Q1. A slightly lower percentage (about 65%) was observed for vaccinees who did not belong to any cohort (Figure 4). Of the 892 vaccinees included at the first dose and 1,879 vaccinees included at the booster dose, 2.2% and 4.7% were immunocompromised, 20.9% and 12.5% had a history of allergy, 15.8% and 12.0% had a prior SARS-CoV-2 infection, 28.1% and 4.0% were children and adolescents, 4.1% and 3.1% were pregnant women, 1.9% and 2.5% were lactating women, and 42.2% and 65.7% did not belong to any of the previous special cohorts (Table 1). Overall, female vaccinees and vaccinees belonging to a special cohort, particularly children and adolescents and adults aged 40–79 years, were more likely to complete the follow-up questionnaires (Table 2). In contrast, a significantly higher proportion of vaccinees not belonging to any special cohort completed only the baseline questionnaire. In terms of vaccine brand and comorbidities, no statistically significant difference was observed between those who completed only the baseline questionnaire and those who completed the baseline questionnaire and Q1, except for those who reported allergies and other diseases. The same pattern was observed when considering subjects who completed up to Q5 (Supplementary Table S1).

**TABLE 1:** Characteristics of vaccinees recruited at the first or booster doses who completed the baseline questionnaire and at least one follow-up questionnaire.

	Immunocompromised		People with a history of allergy		Prior SARS-CoV-2 infection		Children and adolescents		Pregnant women		Lactating women		None of the cohorts	
	First dose N = 20	Booster N = 89	First dose N = 186	Booster N = 235	First dose N = 141	Booster N = 226	First dose N = 251^*^	Booster N = 75*	First dose N = 37	Booster N = 59	First dose N = 17	Booster N = 47	First dose N = 364	Booster N = 1,235
Sex, n (%)														
Males	4 (20.0)	27 (30.3)	64 (34.4)	55 (23.4)	60 (42.6)	86 (38.1)	126 (50.2)	37 (49.3)	0 (0.0)	0 (0.0)	0 (0.0)	0 (0.0)	156 (42.9)	540 (43.7)
Females	16 (80.0)	62 (69.7)	122 (65.6)	180 (76.6)	81 (57.4)	140 (61.9)	125 (49.8)	38 (50.7)	37 (100)	59 (100)	17 (100)	47 (100)	208 (57.1)	695 (56.3)
F/M ratio	4	2.3	1.9	3.3	1.4	1.6	1.0	1.0	-	-	-	-	1.3	1.3
Age category, n (%)														
5–11	1 (5.0)	0 (0.0)	17 (9.1)	1 (0.4)	25 (17.7)	3 (1.3)	134 (53.4)	40 (53.3)	0 (0.0)	0 (0.0)	0 (0.0)	0 (0.0)	0 (0.0)	0 (0.0)
12–17	3 (15.0)	1 (1.1)	25 (13.4)	4 (1.7)	12 (8.5)	7 (3.1)	115 (45.8)	34 (45.3)	0 (0.0)	0 (0.0)	0 (0.0)	0 (0.0)	0 (0.0)	0 (0.0)
18–29	4 (20.0)	8 (9.0)	41 (22.0)	39 (16.6)	24 (17.0)	47 (20.8)	0 (0.0)	0 (0.0)	8 (21.6)	4 (6.8)	0 (0.0)	4 (8.5)	128 (35.2)	211 (17.1)
30–49	9 (45.0)	40 (44.9)	80 (43.0)	109 (46.4)	58 (41.1)	82 (36.3)	0 (0.0)	0 (0.0)	29 (78.4)	55 (93.2)	17 (100)	43 (91.5)	172 (47.3)	521 (42.2)
50–69	3 (15.0)	35 (39.3)	23 (12.4)	74 (31.5)	21 (14.9)	82 (36.3)	0 (0.0)	0 (0.0)	0 (0.0)	0 (0.0)	0 (0.0)	0 (0.0)	59 (16.1)	435 (35.2)
≥70	0 (0.0)	5 (5.6)	0 (0.0)	8 (3.4)	1 (0.7)	5 (2.2)	0 (0.0)	0 (0.0)	0 (0.0)	0 (0.0)	0 (0.0)	0 (0.0)	5 (1.4)	68 (5.5)
COVID-19 vaccination, n (%)														
Comirnaty	17 (85.0)	61 (68.5)	157 (84.4)	144 (61.3)	108 (76.6)	167 (73.9)	238 (94.8)	75 (100)	34 (91.9)	49 (83.1)	14 (82.4)	29 (61.7)	291 (79.9)	719 (58.2)
Jcoven	0 (0.0)	0 (0.0)	0 (0.0)	0 (0.0)	1 (0.7)	0 (0.0)	0 (0.0)	0 (0.0)	0 (0.0)	0 (0.0)	0 (0.0)	0 (0.0)	7 (1.9)	2 (0.2)
Novavax	1 (5.0)	0 (0.0)	1 (0.5)	1 (0.4)	2 (1.4)	0 (0.0)	0 (0.0)	0 (0.0)	0 (0.0)	0 (0.0)	1 (5.9)	1 (2.1)	2 (0.5)	3 (0.2)
Spikevax	2 (10.0)	28 (31.5)	26 (14.0)	88 (37.4)	29 (20.6)	58 (25.7)	13 (5.2)	0 (0.0)	3 (8.1)	10 (16.9)	2 (11.8)	17 (36.2)	58 (15.9)	500 (40.5)
Vaxzevria	0 (0.0)	0 (0.0)	1 (0.5)	1 (0.4)	0 (0.0)	1 (0.4)	0 (0.0)	0 (0.0)	0 (0.0)	0 (0.0)	0 (0.0)	0 (0.0)	2 (0.5)	10 (0.8)
Unknown	0 (0.0)	0 (0.0)	1 (0.5)	1 (0.4)	1 (0.7)	0 (0.0)	0 (0.0)	0 (0.0)	0 (0.0)	0 (0.0)	0 (0.0)	0 (0.0)	4 (1.1)	1 (0.1)
Medical history, n (%)														
Allergy	8 (40.0)	17 (19.1)	186 (100)	235 (100)	29 (20.6)	36 (15.9)	42 (16.7)	5 (6.7)	4 (10.8)	4 (6.8)	3 (17.6)	8 (17.0)	0 (0.0)	0 (0.0)
Cardiovascular disease	3 (15.0)	8 (9)	5 (2.7)	10 (4.3)	6 (4.3)	10 (4.4)	1 (0.4)	0 (0.0)	0 (0.0)	1 (1.7)	0 (0.0)	0 (0.0)	7 (1.9)	47 (3.8)
Diabetes	1 (5.0)	4 (4.5)	1 (0.5)	5 (2.1)	1 (0.7)	7 (3.1)	0 (0.0)	0 (0.0)	0 (0.0)	0 (0.0)	0 (0.0)	0 (0.0)	4 (1.1)	24 (1.9)
Hypertension	2 (10.0)	16 (18)	7 (3.8)	32 (13.6)	10 (7.1)	27 (11.9)	0 (0.0)	0 (0.0)	0 (0.0)	0 (0.0)	0 (0.0)	2 (4.3)	15 (4.1)	150 (12.1)
Immunosuppression	20 (100)	89 (100)	8 (4.3)	17 (7.2)	3 (2.1)	7 (3.1)	4 (1.6)	1 (1.3)	0 (0.0)	1 (1.7)	0 (0.0)	1 (2.1)	0 (0.0)	0 (0.0)
Kidney disease	2 (10.0)	2 (2.2)	6 (3.2)	1 (0.4)	3 (2.1)	1 (0.4)	2 (0.8)	0 (0.0)	0 (0.0)	0 (0.0)	0 (0.0)	0 (0.0)	4 (1.1)	8 (0.6)
Liver disease	2 (10.0)	0 (0.0)	2 (1.1)	1 (0.4)	0 (0.0)	1 (0.4)	0 (0.0)	0 (0.0)	0 (0.0)	0 (0.0)	0 (0.0)	0 (0.0)	2 (0.5)	5 (0.4)
Lung disease	2 (10.0)	7 (7.9)	35 (18.8)	41 (17.4)	10 (7.1)	12 (5.3)	10 (4.0)	2 (2.7)	2 (5.4)	1 (1.7)	1 (5.9)	2 (4.3)	6 (1.6)	30 (2.4)
Malignant tumor	1 (5.0)	10 (11.2)	0 (0.0)	1 (0.4)	2 (1.4)	3 (1.3)	0 (0.0)	0 (0.0)	0 (0.0)	0 (0.0)	0 (0.0)	0 (0.0)	0 (0.0)	9 (0.7)
Neurological disorders	0 (0.0)	5 (5.6)	4 (2.2)	4 (1.7)	1 (0.7)	5 (2.2)	3 (1.2)	0 (0.0)	0 (0.0)	0 (0.0)	0 (0.0)	1 (2.1)	4 (1.1)	13 (1.1)
Pregnancy	0 (0.0)	1 (1.1)	4 (2.2)	4 (1.7)	3 (2.1)	6 (2.7)	0 (0.0)	0 (0.0)	37 (100)	59 (100)	1 (5.9)	0 (0.0)	0 (0.0)	0 (0.0)
Psychological disorders	2 (10.0)	3 (3.4)	7 (3.8)	11 (4.7)	1 (0.7)	8 (3.5)	0 (0.0)	0 (0.0)	0 (0.0)	0 (0.0)	0 (0.0)	0 (0.0)	8 (2.2)	23 (1.9)
Other diseases	6 (30.0)	56 (62.9)	27 (14.5)	59 (25.1)	12 (8.5)	27 (11.9)	21 (8.4)	4 (5.3)	0 (0.0)	8 (13.6)	1 (5.9)	9 (19.1)	52 (14.3)	106 (8.6)

A vaccinee may belong to different cohorts or none of the cohorts, and they may report more than one medical history. A F/M ratio >1 indicates that the number of female participants is higher than the number of male participants. * Children in the age category 0–4 years (N = 2 at the first dose; N = 1 at the booster dose) were also included, even though they were enrolled before the approval of vaccination in children aged between 6 months and 4 years, but they were not considered for the analyses. It is, therefore, likely that these subjects reported an incorrect date of birth when completing the baseline questionnaire.

**TABLE 2:** Characteristics of vaccinees, recruited at the first dose or booster dose, who completed the baseline questionnaire only vs. vaccinees who completed the baseline questionnaire and at least the Q1.

	First vaccination cycle			Booster		
	Baseline N = 379	Baseline + Q1 N = 892*	*p*-value	Baseline N = 451	Baseline + Q1 N = 1,873*	*p*-value
Gender, n (%)						
Males	167 (44.1)	357 (40.0)	0.202	205 (45.5)	727 (38.8)	0.011
Females	212 (55.9)	535 (60.0)		246 (54.5)	1,146 (61.2)	
F/M ratio	1.3	1.5		1.2	1.6	
Age group (y.o.), n (%)						
5–11	21 (5.5)	134 (15.0)	<0.001	14 (3.1)	40 (2.1)	<0.001
12–17	52 (13.7)	115 (12.9)		6 (1.3)	34 (1.8)	
18–39	209 (55.1)	384 (43.0)		210 (46.6)	701 (37.4)	
40–59	81 (21.4)	221 (24.8)		143 (31.7)	791 (42.2)	
60–79	16 (4.2)	36 (4.0)		67 (14.9)	291 (15.5)	
≥80	-	-	-	11 (2.4)	15 (0.8)	
Special cohorts, n (%)						
Immunocompromised	3 (0.8)	20 (2.2)	0.105**	23 (5.1)	89 (4.8)	0.851
People with a history of allergy	58 (15.3)	186 (20.9)	0.026	45 (10)	235 (12.5)	0.154
Prior SARS-CoV-2 infection	53 (14.0)	141 (15.8)	0.458	48 (10.6)	226 (12.1)	0.447
Children and adolescents	73 (19.3)	251 (28.1)	0.001	20 (4.4)	75 (4)	0.778
Pregnant women	9 (2.4)	37 (4.1)	0.141	7 (1.6)	59 (3.2)	0.08
Lactating women	4 (1.1)	17 (1.9)	0.343**	2 (0.4)	47 (2.5)	0.003**
None of these special cohorts	210 (55.4)	364 (40.8)	<0.001	324 (71.8)	1,235 (65.9)	0.019
Vaccine brand, n (%)						
Comirnaty	325 (85.8)	747 (83.7)	0.414	288 (63.9)	1,175 (62.7)	0.697
Jcoven	1 (0.3)	8 (0.9)	0.294**	1 (0.2)	2 (0.1)	0.477**
Novavax	0 (0.0)	5 (0.6)	0.33**	1 (0.2)	4 (0.2)	1**
Spikevax	49 (12.9)	123 (13.8)	0.748	155 (34.4)	678 (36.2)	0.501
Vaxzevria	0 (0.0)	3 (0.3)	0.559**	4 (0.9)	12 (0.6)	0.531**
Unknown	4 (1.1)	6 (0.7)	0.496**	2 (0.4)	2 (0.1)	0.172**
Medical history, n (%)						
No	244 (64.4)	537 (60.2)	0.181	292 (64.7)	1,129 (60.3)	0.090
Yes	135 (35.6)	355 (39.8)		159 (35.3)	744 (39.7)	
Allergy	58 (15.3)	186 (20.9)	0.026	45 (10)	235 (12.5)	0.154
Cardiovascular disease	9 (2.4)	19 (2.1)	0.835	17 (3.8)	70 (3.7)	1
Diabetes	4 (1.1)	7 (0.8)	0.742**	20 (4.4)	36 (1.9)	0.003
Hypertension	15 (4.0)	31 (3.5)	0.743	47 (10.4)	219 (11.7)	0.51
Immunosuppression	3 (0.8)	20 (2.2)	0.105**	23 (5.1)	89 (4.8)	0.851
Kidney disease	4 (1.1)	14 (1.6)	0.609**	8 (1.8)	11 (0.6)	0.019
Liver disease	4 (1.1)	5 (0.6)	0.464**	3 (0.7)	7 (0.4)	0.419**
Lung disease	21 (5.5)	47 (5.6)	0.892	20 (4.4)	80 (4.3)	0.981
Malignant tumor	4 (1.1)	3 (0.3)	0.206**	8 (1.8)	21 (1.1)	0.244
Neurological disorders	8 (2.1)	11 (1.2)	0.311	3 (0.7)	21 (1.1)	0.603**
Pregnancy	9 (2.4)	37 (4.1)	0.141	20 (4.4)	59 (3.2)	0.228
Psychological disorders	9 (2.4)	16 (1.8)	0.511	8 (1.8)	42 (2.2)	0.717
Other diseases	43 (11.3)	102 (11.4)	1	7 (1.6)	240 (12.8)	<0.001

*Children in the age category 0–4 years (N = 2 at the first dose; N = 1 at the booster dose) were also included, even though they were enrolled before the approval of vaccination in children aged between 6 months and 4 years, but not considered for the analyses. It is, therefore, likely that these subjects reported an incorrect date of birth when completing the baseline questionnaire.

**Fisher’s exact test.

**FIGURE 5:**
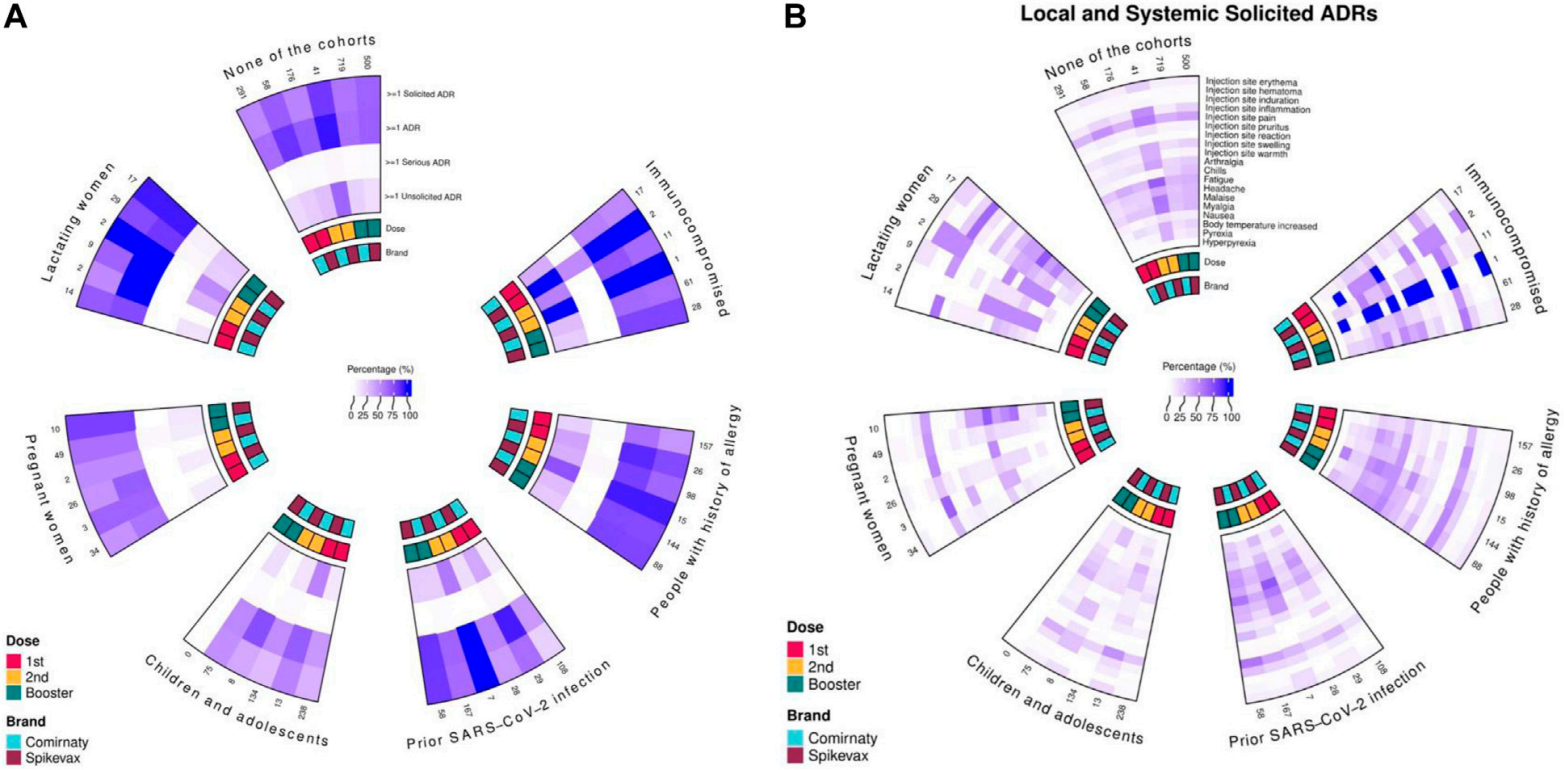
Suspected ADRs reported after receiving a first, second, or booster dose of Comirnaty and Spikevax vaccines by a special cohort. Proportions with at least one, any, solicited, serious, and unsolicited ADRs **(A)** and local and systemic solicited ADRs **(B)**.

Because most participants received the Comirnaty^®^ (69.4%) and, to a lesser extent, the Spikevax^®^ (28.9%) vaccines (Supplementary Table S2), only these brands were retained for the analysis concerning ADR rates. The percentage of vaccinees reporting any ADR following a first, second, or booster dose of the Comirnaty^®^ vaccine was more than 50% across different cohorts (ranging from 51% to 83%) (Figure 5A). However, slightly lower percentages were observed in children and adolescents among different doses (44%–51%), as well as in people with prior SARS-CoV-2 infection after the first dose (43%). Overall, higher rates of ADR were observed after the second dose than after the first dose. Higher percentages of any ADR, ranging from 50% to 100%, were observed following any dose of the Spikevax^®^ vaccine for all the cohorts.

These high percentages included mainly local and systemic solicited ADRs (Figure 5B), with a higher rate of systemic ADRs after the Spikevax^®^ vaccine than the Comirnaty^®^ vaccine. A similar trend was reported for unsolicited ADRs. Overall, the frequency of unsolicited ADRs was higher after the second dose than the first, both for Comirnaty and Spikevax and among all cohorts (Supplementary Table S2). Lymphadenopathy, paresthesia, diarrhea, and vertigo were the most frequently reported unsolicited ADRs (Supplementary Table S3); slightly lower percentages were reported following the booster dose.

Overall, the rate of serious ADRs was, however, low following the first dose (N = 1; 0.1%), the second dose (N = 3; 0.6%), and the booster dose (N = 6; 0.5%) of Comirnaty^®^; higher rates were observed following Spikevax^®^ (N = 2, 1.6%; N = 2, 2.6%; N = 6, 0.9%) (Figure 5A). In general, most of the serious ADRs were related to fever (Supplementary Table S4).

**FIGURE 6:**
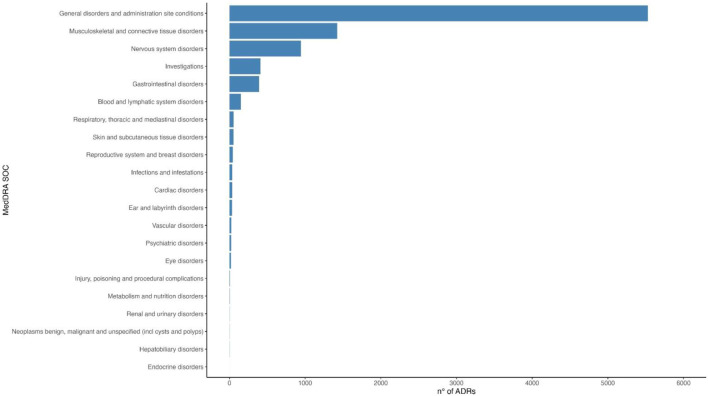
Distribution of all ADRs reported after a first vaccination cycle or a booster dose of Comirnaty and Spikevax vaccines according to MedDRA system organ class (SOC).

Considering all ADRs reported following both the first vaccination cycle and the booster dose, the most frequently reported SOCs (Figure 6) were: 1) General disorders and administration site conditions, 2) Musculoskeletal and connective tissue disorders, 3) Nervous system disorders, 4) Investigations, 5) Gastrointestinal disorders, 6) Blood and lymphatic system disorders, 7) Respiratory, thoracic and mediastinal disorders, and 8) Skin and subcutaneous tissue disorders.

Passive surveillance in Italy recorded 49,237 reports in the first 3 months of the immunization campaign (510 reports per 100,000 doses), 76,206 after 6 months (154 reports per 100,000 administered doses), and 139,622 after 2 years of the immunization campaign (99 reports per 100,000 administered doses) for all COVID-19 vaccines. The percentage of reports with at least one serious reaction were 7%, 11%, and 18%, with a reporting rate of 36, 18, and 18 reports of serious ADR per 100,000 administered doses (from 0.0018% to 0.0036%), respectively. For Comirnaty^®^, reports of serious reactions represented 6%, 9%, and 16% of the overall reports in the first 3 months, 6 months, and 2 years of the immunization campaign, with reporting rates of 33, 14, and 16 reports of serious ADR per 100,000 administered doses (from 0.0014% to 0.0033%), respectively. For Spikevax^®^, reports of serious reactions represented 9%, 16%, and 22% of the overall reports in the first 3 months, first 6 months, and 2 years of the immunization campaign, with reporting rates of 22, 14, and 14 reports of serious ADR per 100,000 administered doses (from 0.00001% to 0.0002%), respectively. The most frequently reported SOCs for all vaccines are in the 2-year reports: 1) General disorders and administration site conditions, 2) Nervous system disorders, 3) Musculoskeletal and connective tissue disorders, 4) Gastrointestinal disorders, 5) Skin and subcutaneous tissue disorders, 6) Blood and lymphatic system disorders, 7) Respiratory, thoracic and mediastinal disorders, and 8) Cardiac disorders.

## 4 Discussion

To the best of our knowledge, this is the first large-scale active surveillance study on COVID-19 vaccines conducted in Italy.

This study confirmed the overall safety profile of mRNA COVID-19 vaccines. The solicited ADRs were expected and well known and were mainly injection site reactions and systemic reactions related to the vaccine immune response, in line with what was observed in pivotal trials (Baden et al., 2021; Polack et al., 2020). Overall, injection site pain was the most commonly reported solicited local ADR among different cohorts and vaccine doses. Fatigue was the most frequently reported systemic ADR following Comirnaty^®^ and Spikevax^®^; high frequencies were also reported for malaise and myalgia following Spikevax^®^. In line with previous observational studies (Chapin-Bardales et al., 2021; Menni et al., 2021; Kant et al., 2022; Salter et al., 2022), differences in reactogenicity between vaccine brands have also been observed in this study, with Spikevax^®^ vaccine being associated with a higher frequency of local and particularly systemic ADRs than Comirnaty^®^, and more frequently after second and booster dose of either vaccine than after the first dose. Although Messer et al. reported that men are more likely to respond to online surveys than women (Messer and Dillman, 2021), in our study, ADRs were more commonly reported by female participants and individuals younger than 59 years than by male participants and those aged 60 years and older, respectively. The trend was similar to that observed in previously published studies (Menni et al., 2021; Amodio et al., 2022; Kant et al., 2022; Rolfes et al., 2022; Meyers et al., 2023). Concerning children and adolescents, lower rates of any ADR (44% at the first dose, 49% at the second dose, and 51% at the booster dose) than adults belonging to other cohorts or no cohorts (<50%) were observed. Accordingly, a previous study measuring the frequency of ADRs after the first and second doses of m-RNA-based COVID-19 vaccines in pediatrics showed that 45% and 40% of pediatric vaccinees reported at least one ADR following the first and the second dose, respectively (Ahmadizar et al., 2023). Furthermore, regarding systemic ADRs specifically, Hause et al. (2021) reported lower rates: 35% and 51% after Comirnaty^®^ first and second doses, respectively. As for vaccinees who reported previous SARS-CoV-2 infection, although it is documented that they may be more likely to experience ADRs than those with no history of infection (Krammer et al., 2021; Manisty et al., 2021), in this study, the percentage of solicited ADRs after a first dose of Comirnaty^®^ was lower than in the other cohorts/no cohorts with lower rates than expected from the published literature (Menni et al., 2021; Kant et al., 2022; Ciccimarra et al., 2024). Consistent with findings from Kant et al. (2022), Raethke et al. (2023), and Rosenblum et al. (2022), and pivotal trials for the general population (Baden et al., 2021; Polack et al., 2020), the frequency of serious ADRs following Comirnaty^®^ was very low among different cohorts/no cohorts and vaccine doses. However, we observed slightly higher rates of severe ADRs after the first and second doses of Spikevax^®^ than in the previously published literature (Kant et al., 2022; Raethke et al., 2023; Rosenblum et al., 2022; Baden et al., 2021), always considering the limited sample size.

Compared with active surveillance performed in this study, passive surveillance recorded significantly lower reporting rates of adverse reactions due to the phenomenon of underreporting. This is true even compared to the first trimester of the immunization campaign when healthcare professionals and the population were likely particularly sensitive to the spontaneous reporting of ADRs. The percentage of serious ADRs is higher for active surveillance than passive surveillance, although the incidence rate remains low. Moreover, the choice of using as denominator subjects completing baseline registration and the Q1 questionnaire could lead to an overestimation of vaccinee-reported ADR rates. Indeed, people dropping out of the study for not completing the Q1 questionnaire are more likely to experience no ADR. Distribution of vaccinee-reported ADRs by SOC is similar for both active and passive surveillance. However, active surveillance appeared to be more sensitive to capturing events belonging to the SOC category “investigations.” This could be explained by an intrinsic difficulty of passive surveillance in collecting alteration of laboratory analyses. Similarly to clinical trials, passive surveillance can rarely provide information on rates of events in special populations like immunocompromised patients. This study was able to collect data in populations of patients where COVID-19 vaccine safety was poorly explored.

Clinical trials conducted before the approval of vaccines play a crucial role in gathering essential information on adverse events following immunization. As the COVID-19 vaccines were introduced and administered to a larger and different population during their rollout, it provided an opportunity to examine the safety profile in a real-world context. Using patient-reported outcomes allows the collection of safety data that may not be documented in medical records (Banerjee et al., 2013). This is especially important for individuals who experience short-term and non-serious ADRs after vaccination and may not consult their physician, thus not contributing to the spontaneous reporting system (Palleria et al., 2013).

As well known, cohort event monitoring studies may be affected by selection bias due to selective non-response (Layton et al., 2015). This bias occurs when individuals who choose not to respond to the study differ significantly from those who do. Selective non-response can lead to an underrepresentation of certain groups within the cohort, potentially skewing the study results. For instance, participants who experienced serious ADRs causing hospitalization may not have been able to complete the follow-up questionnaires, and this may have led to an underestimation of the frequency of serious ADRs, as also reported in previously published literature (Kant et al., 2022). However, cumulative evidence showed that the frequency of serious ADRs was rare (Polack et al., 2020; Kant et al., 2022; Rosenblum et al., 2022; Baden et al., 2021; Raethke et al., 2023).

The involvement of people who contributed to the study was likely higher at the beginning of the vaccination campaign and may have diminished over time, resulting in a loss to follow-up (Raethke et al., 2023; Raethke et al., 2024). Given the 6-month follow-up period, it is unlikely that all participants would complete all questionnaires unless they were highly motivated. In particular, frail participants may have been more likely to complete follow-up questionnaires than healthy participants. Notably, a significantly higher proportion of vaccinees not belonging to any special cohort completed only the baseline questionnaire than vaccinees belonging to a special cohort (55% vs. 41%, *p*-value: <0.001 at the first dose; 72% vs. 66%, *p*-value: 0.019 at the booster dose). Registration to our study could occur up to 2 days after vaccination. This may have introduced a selection bias, as subjects who experienced an ADR shortly after vaccination could be more likely to register. Furthermore, some vaccinee categories, such as older people, may not have had an e-mail address or may have had difficulty using technology. Many of the vaccination centers involved in the study provided dedicated personnel to support vaccinees in participating in the study, specifically facilitating web-based registration to the study and completing the baseline questionnaire, as these were immediate steps after receipt of the vaccine. However, support for completing follow-up questionnaires could not be guaranteed, as questionnaires were sent out at different times in the months following vaccination. In addition, although family members/legal representatives could participate as proxies of the vaccines (e.g., for children or very old vaccinees), issues related to the use of electronic tools (e.g., delivery interruptions or emails being marked as spam) may remain.

The availability of existing networks and infrastructure is crucial for rapid data collection and response. The vaccination campaign started in late December 2020, while in Italy, this study started in June 2021. In other countries, such as the Netherlands, with extensive experience in using the LIM app for data collection (Härmark et al., 2011; van Balveren-Slingerland et al., 2015; Rolfes et al., 2022), the study began in a timely manner in February 2021. In Italy, specifically involved in the safety monitoring of special cohorts, the tools (LIM and RO) had to be adapted according to the different cohorts considered and translated. The lag between the vaccination schedules due to different vaccination strategies and the start of the study may have affected the total number of recruited participants. This issue particularly impacted the recruitment of certain groups, such as immunocompromised individuals, who were vaccinated early in the vaccination strategy and thus were only marginally included in the study.

In addition, variability in data collection methods, definitions, and reporting formats may prevent comparability between different surveillance systems. However, the difficulty of integrating data from different sources was solved through the use of a common data model that allowed data harmonization from the two web apps.

Patient-reported outcomes (PROs) are valuable tools in healthcare, providing insight into patients’ perspectives on their health. However, their accuracy can sometimes be compromised due to several factors, such as missing or incomplete data. The involvement of clinicians could increase the quantity and quality of information collected. In this study, this problem was partly solved by the involvement of qualified and trained monitors in vaccination centers who could support vaccinees in the registration and baseline compilation phases.

Engaging communities in the surveillance process is essential for a timely and successful implementation. In Italy, this has been achieved through the “ilmiovaccinoCOVID19 collaborating group,” a multidisciplinary network of more than 30 partners throughout Italy, including pharmacovigilance experts and clinicians with strong connections with scientific communities.

## 5 Conclusion

Findings from this study confirmed the overall safety information of COVID-19 vaccines. Despite the relatively low number of participants who completed all follow-up questionnaires, our data are in line with those from the pivotal clinical trials and other active surveillance studies. However, higher rates of suspected ADRs and percentages of serious ones were observed compared with passive surveillance, which could be affected by underreporting. CEM studies allow early and near real-time monitoring of drug safety, and this is crucial for regulatory agencies, especially in emergency situations like the COVID-19 pandemic, particularly in populations excluded from pivotal trials. It is of paramount importance to minimize the activation time for such studies by implementing strategies like mock-ups, which involve preparing data collection tools, such as web applications, in advance of vaccination campaigns. The COVID-19 pandemic highlighted the need to invest in infrastructure and systems to ensure a comprehensive and fair assessment of vaccine safety.

Active surveillance studies that involve the systematic collection, analysis, interpretation, and dissemination of safety information face several challenges. Addressing these challenges requires a multidisciplinary approach involving collaboration between public health agencies, researchers, policymakers, and technology experts to improve surveillance infrastructure and methodologies. The availability of consolidated networks that could use vaccine experts, public health infrastructure data, and preexisting relationships is crucial for rapid response and preparedness for future pandemics. This approach could be equally applicable to all vaccination campaigns using innovative vaccines.

## Data Availability

The raw data supporting the conclusions of this article will be made available by the authors, without undue reservation.
