# Supplementary material for: Cohort event monitoring of safety of COVID-19 vaccines: the Italian experience of the “ilmiovaccinoCOVID19 collaborating group”
Source: Front Drug Saf Regul. 2024 Aug 12;4:1363086. doi: 10.3389/fdsfr.2024.1363086 (PMC12445166; doi:10.3389/fdsfr.2024.1363086)
Supplement: Supplementary file 1 [file DataSheet2.PDF]

# Il tuo contributo **e quello di tuo/a figlio/a rendono i vaccini più sicuri**

Fai partecipare tuo/a figlio/a al monitoraggio degli effetti collaterali dei vaccini contro il COVID-19 registrandolo/a al sito web **fino a 48 ore dopo** aver ricevuto **la prima dose di vaccino.**

**COVID** vaccine monitor.eu/it

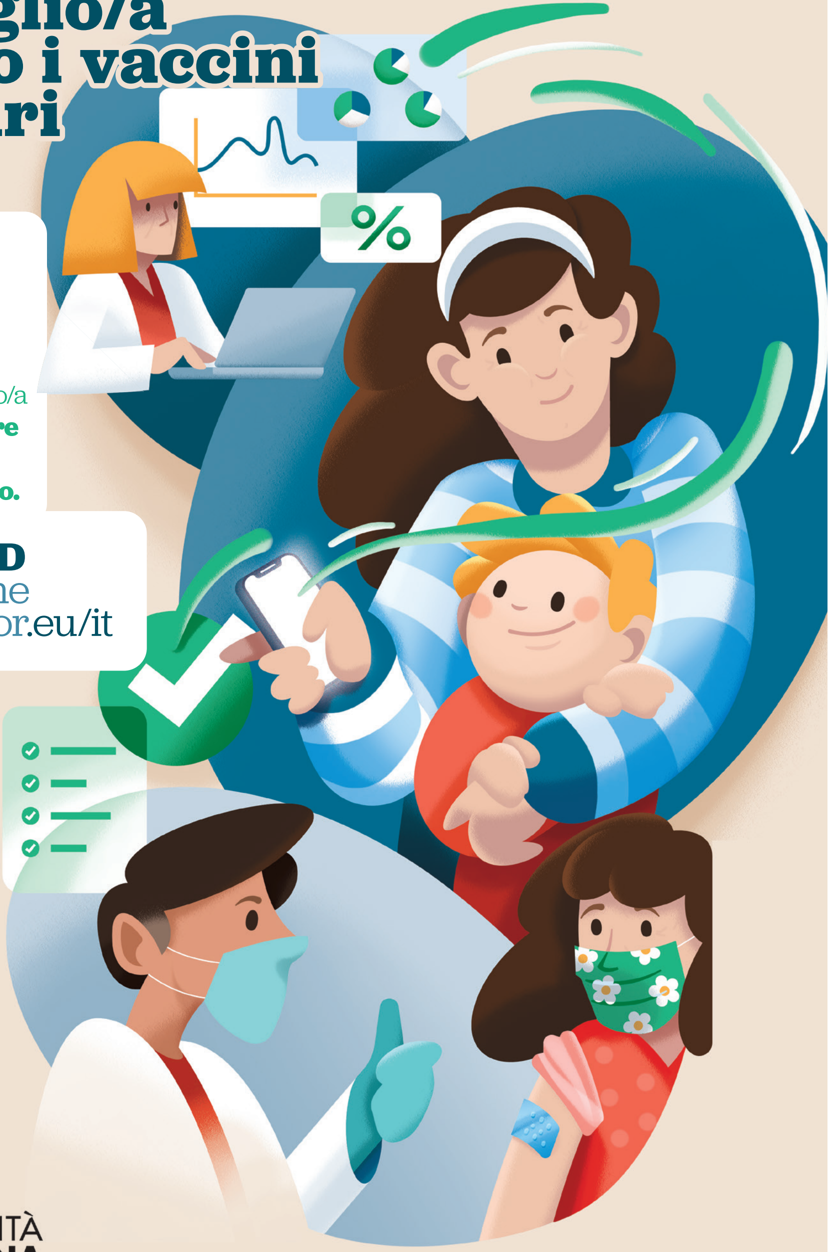


UNIVERSITÀ di **VERONA**

Dipartimento di **DIAGNOSTICA E SANITÀ PUBBLICA**

## **Tuo/a figlio/a ha ricevuto la prima dose di vaccino contro il COVID-19?** Puoi segnalarci qualunque effetto collaterale, è importante!

Questo studio è stato finanziato dall'Agenzia Europea del Farmaco (EMA), responsabile insieme all'Agenzia Italiana del Farmaco (AIFA) dell'approvazione e del monitoraggio post-marketing dei vaccini contro il COVID-19. Il Centro di Coordinamento dell'Università di Verona, insieme ad altri partner italiani ed internazionali (16 Paesi EU e non EU), sta raccogliendo informazioni sui possibili effetti collaterali in seguito alla vaccinazione contro il COVID-19. Fai partecipare anche tuo/a figlio/a!

## Rendere i vaccini **più sicuri**

I vaccini contro il COVID-19 sono stati studiati a fondo e soddisfano tutti i requisiti di sicurezza previsti per qualsiasi altro vaccino. Vista la situazione di emergenza, bambini ed adolescenti sono stati inclusi negli studi clinici più tardi rispetto al resto della popolazione. È dunque fondamentale monitorare con attenzione la loro risposta alla vaccinazione in quanto potrebbero ancora verificarsi effetti collaterali inattesi. Non si sa con quale frequenza questi potrebbero verificarsi o se alcuni bambini/adolescenti hanno maggiori probabilità di sperimentarli.

Sarà in particolare condotto un monitoraggio della risposta alla vaccinazione **nei bambini/adolescenti che ricevono la prima dose di vaccino.** Facendo partecipare tuo/a figlio/a questo studio puoi aiutarci a raccogliere importanti informazioni e rendere l'uso dei vaccini ancora più sicuro. Tutte le informazioni raccolte saranno condivise con l'EMA e l'AIFA e confrontate con quelle degli altri Paesi.

## Partecipare **è semplice**

Tuo/a figlio/a sta per ricevere o ha ricevuto **da non più di 48 ore** la prima dose di vaccino contro il COVID-19? Registralo/a al seguente sito web: **www.covidvaccinemonitor.eu/it**

Sul sito web troverai tutte le informazioni necessarie per partecipare. Dopo la registrazione riceverai una e-mail con le indicazioni per compilare un questionario di base. Riceverai altri sei questionari di follow-up nei sei mesi successivi alla registrazione.

## Cosa **vogliamo sapere**

Nei questionari troverai domande sulla salute di tuo/a figlio/a e sui possibili effetti collaterali che potrebbero essere insorti dopo la vaccinazione. La compilazione di ogni questionario non impegnerà più di 10 minuti. Ricorda che è possibile partecipare **fino a 48 ore** dopo aver ricevuto **la prima dose di vaccino.** Se cambiate idea, potete decidere di ritirarvi ed interrompere la partecipazione allo studio in qualsiasi momento, senza fornire nessuna giustificazione e senza nessuna conseguenza.

**Fai partecipare tuo/a figlio/a allo studio, vai su**

# www.covidvaccinemonitor.eu/it

**e contribuite con noi alla sicurezza dei vaccini contro il COVID-19!**

Questo studio è coordinato dall'Università degli Studi di Verona. I tuoi dati personali e quelli di tuo/a figlio/a saranno trattati con riservatezza.

**CON LA PARTECIPAZIONE DI**

**I loghi dei partner italiani partecipanti al progetto possono essere trovati sul sito: covidvaccinemonitor.eu/it**
