# Supplementary material for: Cohort event monitoring of safety of COVID-19 vaccines: the Italian experience of the “ilmiovaccinoCOVID19 collaborating group”
Source: Front Drug Saf Regul. 2024 Aug 12;4:1363086. doi: 10.3389/fdsfr.2024.1363086 (PMC12445166; doi:10.3389/fdsfr.2024.1363086)
Supplement: Supplementary file 2 [file DataSheet4.PDF]

Il tuo contributo **rende i vaccini più sicuri**

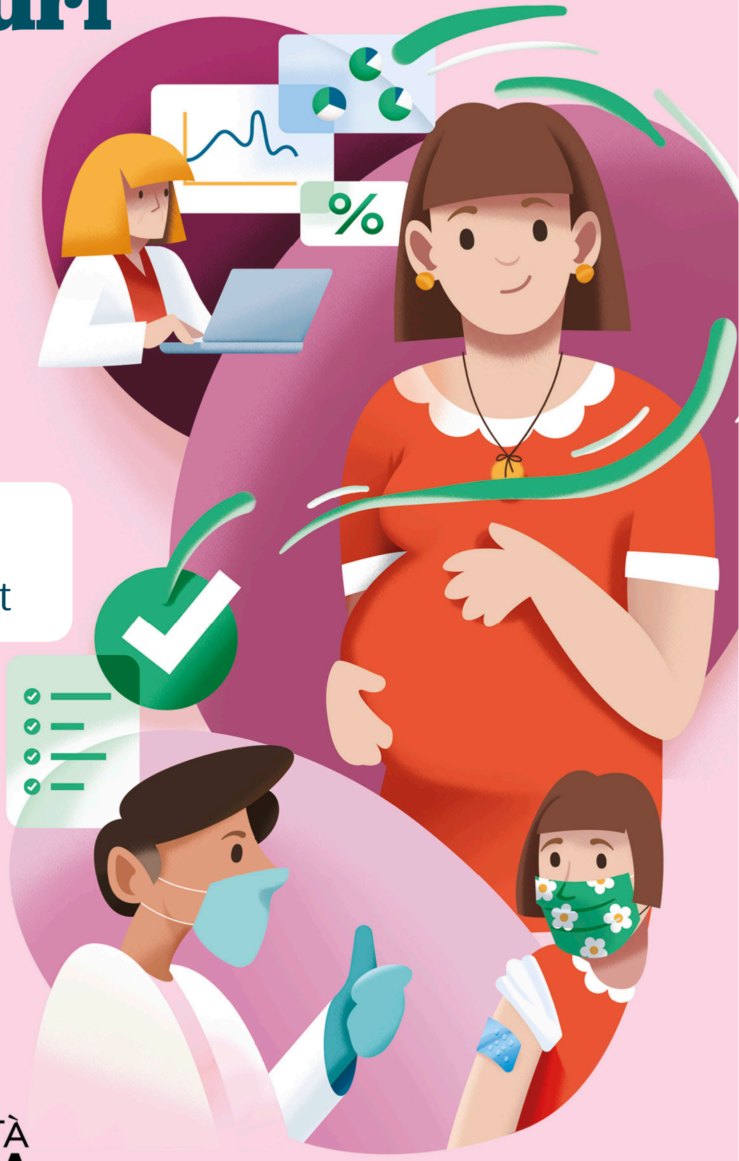

Partecipa al monitoraggio degli effetti collaterali dei vaccini anti COVID-19 registrandoti al sito web **fino a 48 ore dopo aver ricevuto la prima dose del vaccino**.

COVID vaccine monitor.eu/it

UNIVERSITÀ di **VERONA**

Dipartimento di **DIAGNOSTICA E SANITÀ PUBBLICA**

Sei incinta o stai allattando e hai ricevuto la prima dose del vaccino contro il COVID-19?

Puoi segnalarci qualunque effetto collaterale, è importante!

Questo studio è stato finanziato dall'Agenzia Europea del Farmaco (EMA), responsabile insieme all'Agenzia Italiana del Farmaco (AIFA) dell'approvazione e del monitoraggio dei vaccini contro il COVID-19. Il Centro di coordinamento dell'Università di Verona, insieme ad altri partner italiani ed internazionali (16 Paesi EU e non EU), sta raccogliendo informazioni sui possibili effetti collaterali in seguito alla vaccinazione contro il COVID-19. Partecipa anche tu!

Rendere i vaccini ancora più sicuri

I vaccini contro il COVID-19 sono stati studiati a fondo e soddisfano tutti i requisiti di sicurezza previsti per qualsiasi altro vaccino. Inizialmente però, vista la situazione di emergenza, le donne in gravidanza non sono state incluse negli studi clinici ed è molto importante monitorare con attenzione la loro risposta alla vaccinazione. I dati raccolti fino ad oggi nei paesi in cui le donne incinte sono state vaccinate mostrano che i vaccini contro il COVID-19 sono efficaci e sicuri anche in gravidanza.

Partecipando a questo studio puoi aiutarci a raccogliere importanti informazioni anche nel nostro paese per rendere l'uso dei vaccini in gravidanza ancora più sicuro. Tutte le informazioni raccolte saranno condivise con i sistemi di farmacovigilanza di EMA e AIFA e confrontate con quelle degli altri paesi.

Partecipare è semplice

Sei incinta o stai allattando e hai ricevuto da meno di 48 ore la prima dose del vaccino contro il COVID-19? Partecipa allo studio registrandoti al sito web:
covidvaccinemonitor.eu/it

Non importa se la tua gravidanza è appena iniziata o se sei vicina al parto, puoi partecipare qualunque sia la tua epoca gestazionale. Sul sito troverai tutte le informazioni necessarie per partecipare. Dopo la registrazione riceverai una e-mail con le indicazioni per compilare un questionario di base. Poi, nei sei mesi successivi riceverai altri questionari di follow-up. Infine, riceverai un questionario al termine della gravidanza, più o meno dopo 45 giorni dalla data presunta del parto.

Cosa vogliamo sapere

Nei questionari troverai domande sulla tua salute, sulla tua gravidanza e sui possibili effetti collaterali che potresti volerci segnalare dopo la vaccinazione. La compilazione di ogni questionario non impegnerà più di 10 minuti; ricorda che è possibile partecipare **fino a 48 ore dopo la prima dose di vaccino.**

Se dovessi cambiare idea, nessun problema: puoi decidere di ritirarti ed interrompere la partecipazione allo studio in qualsiasi momento, senza bisogno di nessuna giustificazione e senza nessuna conseguenza.

Partecipa allo studio, vai su

covidvaccinemonitor.eu/it

e contribuisci con noi alla sicurezza dei vaccini contro il COVID-19 in gravidanza!

Questo studio è coordinato dall'Università degli Studi di Verona. I tuoi dati personali saranno trattati con riservatezza.

CON LA PARTECIPAZIONE DI

Civico Di Cristina Benfratelli
Regione Campania ASL Caserta
SANI.A.R.P. Campania On Line
Centro Regionale di Farmacovigilanza e Farmacoepidemiologia Regione Campania
Azienda Ospedaliera Universitaria "Gaetano Martino" Messina – Centro Regionale di Farmacovigilanza - Regione Sicilia
ASP Messina
Azienda Ospedale Università Padova
Azienda Ospedaliera Universitaria Integrata Verona
Sacro Cuore Don Calabria IRCCS
Regione Emilia-Romagna – Centro Regionale di FarmacoVigilanza – Direzione Generale Cura della Persona, Salute e Welfare
Servizio Sanitario Regionale Emilia-Romagna – Azienda Unità Sanitaria Locale di Bologna
Istituto delle Scienze Neurologiche – Istituto di Ricovero e Cura a Carattere Scientifico
Regione Emilia-Romagna – Centro Regionale di Farmacovigilanza
Servizio Sanitario Regionale Emilia-Romagna – Azienda Unità Sanitaria Locale di Ferrara
Alma Mater Studiorum Università di Bologna
Azienda Sanitaria Provinciale Reggio Calabria
Università degli Studi di Palermo
A.O.U. Policlinico Paolo Giaccone
INSPIRE
CiaoLapo
Università degli Studi Firenze
Università di Foggia
Regione Toscana
UTIFAR Unione Tecnica Italiana Farmacisti
PEDERZOLI
