# Supplementary material for: Cohort event monitoring of safety of COVID-19 vaccines: the Italian experience of the “ilmiovaccinoCOVID19 collaborating group”
Source: Front Drug Saf Regul. 2024 Aug 12;4:1363086. doi: 10.3389/fdsfr.2024.1363086 (PMC12445166; doi:10.3389/fdsfr.2024.1363086)
Supplement: Supplementary file 3 [file Table1.DOCX]

**Supplementary table 1.** Characteristics of vaccinees, recruited at first dose or booster dose, who filled the baseline questionnaire only vs. vaccinees who filled the baseline questionnaire and the follow-up questionnaires up to Q5.

|  | **First vaccination cycle** | | | **Booster** | | |
| --- | --- | --- | --- | --- | --- | --- |
|  | **Baseline**  **N= 379** | **Baseline + Q5**  **N= 354*** | **p-value** | **Baseline**  **N= 451** | **Baseline + Q5**  **N= 606** | **p-value** |
| **Gender, n (%)** |  | | |  | | |
| Males | 167 (44.1) | 140 (39.5) | 0.245 | 205 (45.5) | 236 (38.9) | 0.0394 |
| Females | 212 (55.9) | 214 (64.5) |  | 246 (54.5) | 370 (61.1) |  |
| F/M ratio | 1.3 | 1.5 |  | 1.2 | 1.6 |  |
| **Age group (y.o.), n (%)** |  | | |  | | |
| 5-11 | 21 (5.5) | 51 (14.4) | <0.001 | 14 (3.1) | 14 (2.3) | <0.001 |
| 12-17 | 52 (13.7) | 56 (15.8) |  | 6 (1.3) | 16 (2.6) |  |
| 18-39 | 209 (55.1) | 131 (37.0) |  | 210 (46.6) | 224 (37.0) |  |
| 40-59 | 81 (21.4) | 101 (28.5) |  | 143 (31.7) | 250 (41.3) |  |
| 60-79 | 16 (4.2) | 13 (3.7) |  | 67 (14.9) | 98 (16.2) |  |
| ≥80 | - | - | - | 11 (2.4) | 4 (0.7) |  |
| **Special cohorts, n (%)** |  | | |  | | |
| Immunocompromised | 3 (0.8) | 10 (2.8) | 0.071 | 23 (5.1) | 26 (4.3) | 0.638 |
| People with history of allergy | 58 (15.3) | 81 (22.9) | 0.012 | 45 (10) | 86 (14.2) | 0.050 |
| Prior SARS-CoV-2 infection | 53 (14.0) | 49 (13.8) | 1.000 | 48 (10.6) | 74 (12.2) | 0.489 |
| Children and adolescents | 73 (19.3) | 107 (30.2) | 0.001 | 20 (4.4) | 30 (5.0) | 0.807 |
| Pregnant women | 9 (2.4) | 6 (1.7) | 0.698 | 7 (1.6) | 23 (3.8) | 0.047 |
| Lactating women | 4 (1.1) | 7 (2.0) | 0.470 | 2 (0.4) | 20 (3.3) | 0.003 |
| None of these special cohorts | 210 (55.4) | 133 (37.6) | <0.001 | 324 (71.8) | 383 (63.2) | 0.004 |
| **Vaccine brand, n (%)** |  | | |  | | |
| Comirnaty | 325 (85.8) | 295 (83.3) | 0.421 | 288 (63.9) | 329 (54.3) | 0.002 |
| Jcoven | 1 (0.3) | 1 (0.3) | 1.000 | 1 (0.2) | 0 (0.0) | - |
| Novavax | 0 (0.0) | 1 (0.3) | - | 1 (0.2) | 2 (0.3) | 1.000 |
| Spikevax | 49 (12.9) | 54 (15.3) | 0.424 | 155 (34.4) | 272 (44.9) | 0.411 |
| Vaxzevria | 0 (0.0) | 0 (0.0) | - | 4 (0.9) | 2 (0.3) | 0.411 |
| Unknown | 4 (1.1) | 3 (0.8) | 1.000 | 2 (0.4) | 1 (0.2) | 0.579 |
| **Medical history, n (%)** |  | | |  | | |
| No | 244 (64.4) | 207 (58.5) | 0.117 | 292 (64.7) | 359 (59.2) | 0.079 |
| Yes | 135 (35.6) | 147 (41.5) |  | 159 (35.3) | 247 (40.8) |  |
| Allergy | 58 (15.3) | 81 (22.9) | 0.012 | 45 (10) | 86 (14.0) | 0.050 |
| Cardiovascular disease | 9 (2.4) | 6 (1.7) | 0.698 | 17 (3.8) | 20 (3.3) | 0.809 |
| Diabetes | 4 (1.1) | 2 (0.6) | 0.687 | 20 (4.4) | 6 (1.0) | <0.001 |
| Hypertension | 15 (4.0) | 7 (2.0) | 0.176 | 47 (10.4) | 74 (12.2) | 0.420 |
| Immunosuppression | 3 (0.8) | 10 (2.8) | 0.049 | 23 (5.1) | 26 (4.3) | 0.638 |
| Kidney disease | 4 (1.1) | 3 (0.8) | 1.000 | 8 (1.8) | 2 (0.3) | 0.022 |
| Liver disease | 4 (1.1) | 1 (0.3) | 0.375 | 3 (0.7) | 5 (0.8) | 1.000 |
| Lung disease | 21 (5.5) | 24 (6.8) | 0.663 | 20 (4.4) | 24 (4.0) | 0.821 |
| Malignant tumor | 4 (1.1) | 1 (0.3) | 0.698 | 8 (1.8) | 6 (1.0) | 0.406 |
| Neurological disorders | 8 (2.1) | 5 (1.4) | 0.663 | 3 (0.7) | 7 (1.2) | 0.530 |
| Pregnancy | 9 (2.4) | 6 (1.7) | 0.698 | 20 (4.4) | 23 (3.8) | 0.717 |
| Psychological disorders | 9 (2.4) | 9 (2.5) | 1.000 | 8 (1.8) | 14 (2.3) | 0.699 |
| Other diseases | 43 (11.3) | 54 (15.3) | 0.147 | 7 (1.6) | 75 (12.4) | <0.001 |
| *Note: one vaccinee may belong to different cohorts.*  **Children in the age category 0-4 years (N=2 at the first dose) were also included, even though they were enrolled before the approval of vaccination in children aged between 6 months and 4 years, but not considered for the analyses. It is therefore likely that these subjects reported an incorrect date of birth when completing the baseline questionnaire* | | | | | | |

**Supplementary table 2.** Suspected ADRs reported after receiving a first, second or booster dose of Comirnaty and Spikevax vaccines, by special cohort.

|  | **Immunocompromised** | | | **People with**  **history of allergy** | | | **Prior SARS-CoV-2**  **infection** | | | **Children and**  **adolescents** | | | **Pregnant women** | | | **Lactating women** | | | **None of the cohorts** | | |
| --- | --- | --- | --- | --- | --- | --- | --- | --- | --- | --- | --- | --- | --- | --- | --- | --- | --- | --- | --- | --- | --- |
|  | **1^st^ dose** | **2^nd^ dose** | **Booster dose** | **1^st^ dose** | **2^nd^ dose** | **Booster dose** | **1^st^ dose** | **2^nd^ dose** | **Booster dose** | **1^st^ dose** | **2^nd^ dose** | **Booster dose** | **1^st^ dose** | **2^nd^ dose** | **Booster dose** | **1^st^ dose** | **2^nd^ dose** | **Booster dose** | **1^st^ dose** | **2^nd^ dose** | **Booster dose** |
| **Comirnaty** |  | | |  | | |  | | |  | | |  | | |  | | |  | | |
| **N. of vaccinees** | **17** | **11** | **61** | **157** | **98** | **144** | **108** | **28** | **167** | **238** | **134** | **75** | **34** | **26** | **49** | **14** | **9** | **29** | **291** | **176** | **719** |
| **≥1 solicited ADR, n (%)** | 10 (50) | 7 (63.6) | 38 (62.3) | 83 (44.6) | 64 (65.3) | 110 (76.4) | 28 (19.9) | 12 (42.9) | 106 (63.5) | 83 (33.1) | 37 (27.6) | 36 (48) | 17 (45.9) | 12 (46.2) | 30 (61.2) | 12 (70.6) | 6 (66.7) | 22 (75.9) | 148 (50.9) | 94 (53.4) | 426 (59.2) |
| **Local solicited ADRs, n (%)** |  | | |  | | |  | | |  | | |  | | |  | | |  | | |
| Injection site erythema | 2 (10) | 1 (9.1) | 2 (3.3) | 5 (2.7) | 2 (2) | 14 (9.7) | 1 (0.7) | 1 (3.6) | 9 (5.4) | 4 (1.6) | 0 (0) | 4 (5.3) | 2 (5.4) | 1 (3.8) | 1 (2) | 0 (0) | 1 (11.1) | 2 (6.9) | 12 (4.1) | 12 (6.8) | 39 (5.4) |
| Injection site hematoma | 0 (0) | 1 (9.1) | 0 (0) | 4 (2.2) | 3 (3.1) | 2 (1.4) | 0 (0) | 0 (0) | 2 (1.2) | 2 (0.8) | 0 (0) | 1 (1.3) | 0 (0) | 0 (0) | 1 (2) | 0 (0) | 1 (11.1) | 0 (0) | 3 (1) | 4 (2.3) | 10 (1.4) |
| Injection site induration | 0 (0) | 0 (0) | 0 (0) | 1 (0.5) | 0 (0) | 1 (0.7) | 0 (0) | 0 (0) | 1 (0.6) | 0 (0) | 0 (0) | 0 (0) | 0 (0) | 0 (0) | 0 (0) | 0 (0) | 0 (0) | 0 (0) | 2 (0.7) | 2 (1.1) | 4 (0.6) |
| Injection site inflammation | 3 (15) | 3 (27.3) | 5 (8.2) | 9 (4.8) | 10 (10.2) | 27 (18.8) | 4 (2.8) | 1 (3.6) | 28 (16.8) | 10 (4) | 4 (3) | 9 (12) | 0 (0) | 2 (7.7) | 3 (6.1) | 0 (0) | 0 (0) | 6 (20.7) | 26 (8.9) | 19 (10.8) | 56 (7.8) |
| Injection site pain | 7 (35) | 6 (54.5) | 27 (44.3) | 56 (30.1) | 38 (38.8) | 73 (50.7) | 19 (13.5) | 6 (21.4) | 65 (38.9) | 59 (23.5) | 24 (17.9) | 25 (33.3) | 11 (29.7) | 6 (23.1) | 22 (44.9) | 10 (58.8) | 4 (44.4) | 18 (62.1) | 89 (30.6) | 58 (33) | 241 (33.5) |
| Injection site pruritus | 0 (0) | 0 (0) | 3 (4.9) | 3 (1.6) | 0 (0) | 10 (6.9) | 4 (2.8) | 2 (7.1) | 3 (1.8) | 3 (1.2) | 1 (0.7) | 1 (1.3) | 1 (2.7) | 2 (7.7) | 0 (0) | 0 (0) | 0 (0) | 2 (6.9) | 10 (3.4) | 9 (5.1) | 27 (3.8) |
| Injection site reaction | 0 (0) | 0 (0) | 1 (1.6) | 0 (0) | 0 (0) | 1 (0.7) | 0 (0) | 0 (0) | 1 (0.6) | 0 (0) | 0 (0) | 0 (0) | 2 (5.4) | 0 (0) | 0 (0) | 1 (5.9) | 0 (0) | 0 (0) | 1 (0.3) | 1 (0.6) | 4 (0.6) |
| Injection site swelling | 4 (20) | 3 (27.3) | 5 (8.2) | 10 (5.4) | 10 (10.2) | 28 (19.4) | 5 (3.5) | 0 (0) | 23 (13.8) | 14 (5.6) | 1 (0.7) | 8 (10.7) | 3 (8.1) | 3 (11.5) | 4 (8.2) | 1 (5.9) | 1 (11.1) | 8 (27.6) | 28 (9.6) | 21 (11.9) | 87 (12.1) |
| Injection site warmth | 2 (10) | 2 (18.2) | 3 (4.9) | 4 (2.2) | 6 (6.1) | 9 (6.3) | 2 (1.4) | 1 (3.6) | 15 (9) | 1 (0.4) | 3 (2.2) | 3 (4) | 1 (2.7) | 2 (7.7) | 1 (2) | 1 (5.9) | 0 (0) | 2 (6.9) | 11 (3.8) | 12 (6.8) | 14 (1.9) |
| **Systemic solicited ADRs, n (%)** |  | | |  | | |  | | |  | | |  | | |  | | |  | | |
| Arthralgia | 3 (15) | 2 (18.2) | 11 (18) | 19 (10.2) | 21 (21.4) | 39 (27.1) | 2 (1.4) | 2 (7.1) | 27 (16.2) | 9 (3.6) | 6 (4.5) | 5 (6.7) | 1 (2.7) | 2 (7.7) | 5 (10.2) | 1 (5.9) | 1 (11.1) | 5 (17.2) | 22 (7.6) | 23 (13.1) | 118 (16.4) |
| Chills | 1 (5) | 0 (0) | 11 (18) | 14 (7.5) | 3 (3.1) | 43 (29.9) | 6 (4.3) | 1 (3.6) | 44 (26.3) | 4 (1.6) | 0 (0) | 5 (6.7) | 0 (0) | 2 (7.7) | 4 (8.2) | 0 (0) | 1 (11.1) | 10 (34.5) | 13 (4.5) | 2 (1.1) | 128 (17.8) |
| Fatigue | 5 (25) | 5 (45.5) | 22 (36.1) | 50 (26.9) | 43 (43.9) | 66 (45.8) | 18 (12.8) | 6 (21.4) | 62 (37.1) | 35 (13.9) | 20 (14.9) | 24 (32) | 8 (21.6) | 5 (19.2) | 10 (20.4) | 5 (29.4) | 1 (11.1) | 11 (37.9) | 71 (24.4) | 51 (29) | 216 (30) |
| Headache | 3 (15) | 3 (27.3) | 15 (24.6) | 28 (15.1) | 26 (26.5) | 52 (36.1) | 12 (8.5) | 4 (14.3) | 36 (21.6) | 25 (10) | 17 (12.7) | 19 (25.3) | 1 (2.7) | 4 (15.4) | 7 (14.3) | 4 (23.5) | 1 (11.1) | 9 (31) | 43 (14.8) | 35 (19.9) | 180 (25) |
| Malaise | 4 (20) | 3 (27.3) | 14 (23) | 31 (16.7) | 28 (28.6) | 48 (33.3) | 10 (7.1) | 5 (17.9) | 50 (29.9) | 14 (5.6) | 13 (9.7) | 14 (18.7) | 4 (10.8) | 4 (15.4) | 7 (14.3) | 3 (17.6) | 2 (22.2) | 6 (20.7) | 35 (12) | 36 (20.5) | 190 (26.4) |
| Myalgia | 4 (20) | 6 (54.5) | 19 (31.1) | 36 (19.4) | 29 (29.6) | 54 (37.5) | 9 (6.4) | 3 (10.7) | 46 (27.5) | 17 (6.8) | 8 (6) | 12 (16) | 2 (5.4) | 4 (15.4) | 6 (12.2) | 2 (11.8) | 0 (0) | 7 (24.1) | 47 (16.2) | 43 (24.4) | 178 (24.8) |
| Nausea | 2 (10) | 1 (9.1) | 8 (13.1) | 18 (9.7) | 13 (13.3) | 23 (16) | 6 (4.3) | 3 (10.7) | 17 (10.2) | 5 (2) | 6 (4.5) | 6 (8) | 1 (2.7) | 0 (0) | 6 (12.2) | 1 (5.9) | 0 (0) | 0 (0) | 17 (5.8) | 11 (6.3) | 54 (7.5) |
| Body temperature increased | 1 (5) | 2 (18.2) | 3 (4.9) | 2 (1.1) | 14 (14.3) | 22 (15.3) | 5 (3.5) | 1 (3.6) | 19 (11.4) | 3 (1.2) | 7 (5.2) | 7 (9.3) | 1 (2.7) | 2 (7.7) | 4 (8.2) | 0 (0) | 1 (11.1) | 3 (10.3) | 7 (2.4) | 18 (10.2) | 86 (12) |
| Pyrexia | 1 (5) | 2 (18.2) | 6 (9.8) | 5 (2.7) | 7 (7.1) | 14 (9.7) | 2 (1.4) | 2 (7.1) | 21 (12.6) | 4 (1.6) | 6 (4.5) | 6 (8) | 0 (0) | 0 (0) | 1 (2) | 0 (0) | 0 (0) | 4 (13.8) | 3 (1) | 7 (4) | 51 (7.1) |
| Hyperpyrexia | 0 (0) | 0 (0) | 0 (0) | 0 (0) | 0 (0) | 0 (0) | 0 (0) | 0 (0) | 1 (0.6) | 0 (0) | 0 (0) | 0 (0) | 0 (0) | 0 (0) | 0 (0) | 0 (0) | 0 (0) | 0 (0) | 0 (0) | 0 (0) | 0 (0) |
| **≥1 ADR, n (%)** | 11 (55) | 8 (72.7) | 39 (63.9) | 114 (61.3) | 79 (80.6) | 112 (77.8) | 61 (43.3) | 15 (53.6) | 111 (66.5) | 110 (43.8) | 66 (49.3) | 38 (50.7) | 22 (59.5) | 18 (69.2) | 30 (61.2) | 13 (76.5) | 9 (100) | 24 (82.8) | 180 (61.9) | 124 (70.5) | 445 (61.9) |
| **≥1 serious ADRs n (%)** | 0 (0) | 0 (0) | 0 (0) | 1 (0.5) | 2 (2) | 0 (0) | 0 (0) | 0 (0) | 1 (0.6) | 0 (0) | 1 (0.7) | 1 (1.3) | 0 (0) | 0 (0) | 1 (2) | 0 (0) | 0 (0) | 1 (3.4) | 0 (0) | 1 (0.6) | 2 (0.3) |
| **≥1 unsolicited ADR, n (%)** | 7 (35) | 6 (54.5) | 17 (27.9) | 36 (19.4) | 34 (34.7) | 40 (27.8) | 12 (8.5) | 4 (14.3) | 35 (21) | 25 (10) | 15 (11.2) | 10 (13.3) | 3 (8.1) | 2 (7.7) | 4 (8.2) | 2 (11.8) | 3 (33.3) | 6 (20.7) | 47 (16.2) | 48 (27.3) | 137 (19.1) |
| **Spikevax** |  | | |  | | |  | | |  | | |  | | |  | | |  | | |
| **N. of vaccinees** | **2** | **1** | **28** | **26** | **15** | **88** | **29** | **7** | **58** | **13** | **8** | **0** | **3** | **2** | **10** | **2** | **2** | **17** | **58** | **41** | **500** |
| **≥1 solicited ADR, n (%)** | 2 (100) | 1 (100) | 22 (78.6) | 20 (76.9) | 14 (93.3) | 69 (78.4) | 18 (62.1) | 7 (100) | 48 (82.8) | 7 (53.8) | 3 (37.5) | - | 2 (66.7) | 1 (50) | 8 (80) | 1 (50) | 2 (100) | 16 (94.1) | 40 (69) | 34 (82.9) | 335 (67) |
| **Local solicited ADRs, n (%)** |  | | |  | | |  | | |  | | |  | | |  | | |  | | |
| Injection site erythema | 1 (50) | 1 (100) | 2 (7.1) | 1 (3.8) | 1 (6.7) | 10 (11.4) | 2 (6.9) | 1 (14.3) | 4 (6.9) | 2 (15.4) | 1 (12.5) | - | 0 (0) | 0 (0) | 0 (0) | 0 (0) | 0 (0) | 3 (17.6) | 6 (10.3) | 8 (19.5) | 33 (6.6) |
| Injection site hematoma | 0 (0) | 0 (0) | 1 (3.6) | 1 (3.8) | 0 (0) | 3 (3.4) | 2 (6.9) | 1 (14.3) | 2 (3.4) | 0 (0) | 0 (0) | - | 0 (0) | 0 (0) | 0 (0) | 0 (0) | 1 (50) | 3 (17.6) | 1 (1.7) | 0 (0) | 7 (1.4) |
| Injection site induration | 0 (0) | 0 (0) | 1 (3.6) | 0 (0) | 0 (0) | 1 (1.1) | 0 (0) | 0 (0) | 0 (0) | 1 (7.7) | 0 (0) | - | 0 (0) | 0 (0) | 0 (0) | 0 (0) | 1 (50) | 0 (0) | 1 (1.7) | 1 (2.4) | 2 (0.4) |
| Injection site inflammation | 1 (50) | 0 (0) | 5 (17.9) | 1 (3.8) | 3 (20) | 24 (27.3) | 3 (10.3) | 1 (14.3) | 15 (25.9) | 2 (15.4) | 2 (25) | - | 0 (0) | 0 (0) | 1 (10) | 0 (0) | 1 (50) | 3 (17.6) | 10 (17.2) | 20 (48.8) | 95 (19) |
| Injection site pain | 1 (50) | 1 (100) | 16 (57.1) | 12 (46.2) | 7 (46.7) | 47 (53.4) | 9 (31) | 2 (28.6) | 34 (58.6) | 6 (46.2) | 2 (25) | - | 2 (66.7) | 1 (50) | 4 (40) | 0 (0) | 1 (50) | 11 (64.7) | 27 (46.6) | 23 (56.1) | 198 (39.6) |
| Injection site pruritus | 1 (50) | 0 (0) | 1 (3.6) | 0 (0) | 0 (0) | 4 (4.5) | 2 (6.9) | 0 (0) | 1 (1.7) | 0 (0) | 1 (12.5) | - | 0 (0) | 0 (0) | 0 (0) | 0 (0) | 0 (0) | 2 (11.8) | 4 (6.9) | 5 (12.2) | 23 (4.6) |
| Injection site reaction | 0 (0) | 0 (0) | 0 (0) | 0 (0) | 0 (0) | 1 (1.1) | 0 (0) | 0 (0) | 0 (0) | 0 (0) | 0 (0) | - | 0 (0) | 0 (0) | 0 (0) | 0 (0) | 0 (0) | 0 (0) | 0 (0) | 0 (0) | 1 (0.2) |
| Injection site swelling | 1 (50) | 1 (100) | 5 (17.9) | 4 (15.4) | 2 (13.3) | 26 (29.5) | 5 (17.2) | 0 (0) | 17 (29.3) | 1 (7.7) | 2 (25) | - | 1 (33.3) | 0 (0) | 0 (0) | 0 (0) | 0 (0) | 4 (23.5) | 8 (13.8) | 14 (34.1) | 98 (19.6) |
| Injection site warmth | 0 (0) | 1 (100) | 3 (10.7) | 0 (0) | 3 (20) | 13 (14.8) | 1 (3.4) | 1 (14.3) | 6 (10.3) | 2 (15.4) | 2 (25) | - | 0 (0) | 0 (0) | 1 (10) | 0 (0) | 0 (0) | 1 (5.9) | 5 (8.6) | 14 (34.1) | 25 (5) |
| **Systemic solicited ADRs, n (%)** |  | | |  | | |  | | |  | | |  | | |  | | |  | | |
| Arthralgia | 0 (0) | 1 (100) | 12 (42.9) | 3 (11.5) | 7 (46.7) | 23 (26.1) | 5 (17.2) | 3 (42.9) | 18 (31) | 1 (7.7) | 0 (0) | - | 0 (0) | 0 (0) | 4 (40) | 1 (50) | 0 (0) | 5 (29.4) | 6 (10.3) | 16 (39) | 107 (21.4) |
| Chills | 0 (0) | 0 (0) | 10 (35.7) | 2 (7.7) | 3 (20) | 30 (34.1) | 5 (17.2) | 3 (42.9) | 19 (32.8) | 1 (7.7) | 0 (0) | - | 0 (0) | 0 (0) | 4 (40) | 1 (50) | 1 (50) | 7 (41.2) | 2 (3.4) | 1 (2.4) | 135 (27) |
| Fatigue | 2 (100) | 1 (100) | 14 (50) | 11 (42.3) | 7 (46.7) | 45 (51.1) | 11 (37.9) | 4 (57.1) | 34 (58.6) | 1 (7.7) | 1 (12.5) | - | 1 (33.3) | 1 (50) | 7 (70) | 1 (50) | 0 (0) | 7 (41.2) | 21 (36.2) | 28 (68.3) | 170 (34) |
| Headache | 1 (50) | 0 (0) | 5 (17.9) | 5 (19.2) | 7 (46.7) | 38 (43.2) | 9 (31) | 5 (71.4) | 22 (37.9) | 3 (23.1) | 1 (12.5) | - | 1 (33.3) | 0 (0) | 4 (40) | 1 (50) | 1 (50) | 12 (70.6) | 12 (20.7) | 15 (36.6) | 155 (31) |
| Malaise | 1 (50) | 1 (100) | 13 (46.4) | 9 (34.6) | 8 (53.3) | 39 (44.3) | 13 (44.8) | 3 (42.9) | 26 (44.8) | 3 (23.1) | 1 (12.5) | - | 0 (0) | 0 (0) | 5 (50) | 1 (50) | 1 (50) | 7 (41.2) | 10 (17.2) | 24 (58.5) | 150 (30) |
| Myalgia | 1 (50) | 1 (100) | 9 (32.1) | 11 (42.3) | 7 (46.7) | 37 (42) | 11 (37.9) | 3 (42.9) | 24 (41.4) | 4 (30.8) | 0 (0) | - | 0 (0) | 0 (0) | 6 (60) | 1 (50) | 1 (50) | 9 (52.9) | 14 (24.1) | 23 (56.1) | 156 (31.2) |
| Nausea | 0 (0) | 0 (0) | 6 (21.4) | 5 (19.2) | 3 (20) | 16 (18.2) | 4 (13.8) | 2 (28.6) | 11 (19) | 1 (7.7) | 0 (0) | - | 0 (0) | 0 (0) | 1 (10) | 1 (50) | 1 (50) | 4 (23.5) | 1 (1.7) | 5 (12.2) | 40 (8) |
| Body temperature increased | 2 (100) | 0 (0) | 5 (17.9) | 4 (15.4) | 3 (20) | 18 (20.5) | 2 (6.9) | 1 (14.3) | 16 (27.6) | 2 (15.4) | 2 (25) | - | 0 (0) | 0 (0) | 1 (10) | 0 (0) | 0 (0) | 4 (23.5) | 8 (13.8) | 14 (34.1) | 90 (18) |
| Pyrexia | 0 (0) | 1 (100) | 5 (17.9) | 3 (11.5) | 8 (53.3) | 24 (27.3) | 7 (24.1) | 2 (28.6) | 12 (20.7) | 1 (7.7) | 2 (25) | - | 0 (0) | 0 (0) | 2 (20) | 0 (0) | 0 (0) | 3 (17.6) | 1 (1.7) | 14 (34.1) | 56 (11.2) |
| Hyperpyrexia | 0 (0) | 0 (0) | 0 (0) | 0 (0) | 0 (0) | 0 (0) | 1 (3.4) | 0 (0) | 0 (0) | 0 (0) | 0 (0) | - | 0 (0) | 0 (0) | 0 (0) | 0 (0) | 0 (0) | 0 (0) | 0 (0) | 0 (0) | 1 (0.2) |
| **≥1 ADR, n (%)** | 2 (100) | 1 (100) | 22 (78.6) | 23 (88.5) | 14 (93.3) | 70 (79.5) | 27 (93.1) | 7 (100) | 49 (84.5) | 9 (69.2) | 6 (75) | - | 2 (66.7) | 1 (50) | 8 (80) | 2 (100) | 2 (100) | 16 (94.1) | 49 (84.5) | 39 (95.1) | 341 (68.2) |
| **≥1 serious ADR, n (%)** | 1 (50) | 0 (0) | 0 (0) | 0 (0) | 0 (0) | 1 (1.1) | 1 (3.4) | 0 (0) | 0 (0) | 0 (0) | 0 (0) | - | 0 (0) | 0 (0) | 0 (0) | 0 (0) | 0 (0) | 1 (5.9) | 1 (1.7) | 1 (2.4) | 5 (1) |
| **≥1 unsolicited ADR, n (%)** | 2 (100) | 1 (100) | 6 (21.4) | 10 (38.5) | 11 (73.3) | 20 (22.7) | 11 (37.9) | 4 (57.1) | 12 (20.7) | 7 (53.8) | 0 (0) | - | 0 (0) | 0 (0) | 1 (10) | 0 (0) | 1 (50) | 4 (23.5) | 12 (20.7) | 27 (65.9) | 67 (13.4) |

**Supplementary table 3.** List of the overall reported unsolicited ADRs (MedDRA Preferred Terms), following a first, second or booster dose of any COVID-19 vaccine.

|  | **First dose** | **Second dose** | **Booster dose** |
| --- | --- | --- | --- |
| Lymphadenopathy | 26 | 19 | 110 |
| Paraesthesia | 17 | 12 | 11 |
| Diarrhoea | 16 | 11 | 17 |
| Vertigo | 14 | 12 | 5 |
| Extensive swelling of vaccinated limb | 9 | 4 | - |
| Abdominal pain | 7 | 5 | 5 |
| Oropharyngeal pain | 7 | 5 | 5 |
| Pain in extremity | 7 | 6 | 12 |
| Cough | 6 | 4 | 4 |
| Tinnitus | 6 | 5 | 2 |
| Urticaria | 6 | 5 | 1 |
| Feeling hot | 5 | 2 | 1 |
| Menstruation irregular | 5 | 5 | 4 |
| Nasopharyngitis | 5 | 2 | 9 |
| Tachycardia | 5 | 3 | 14 |
| Amenorrhoea | 4 | 4 | - |
| Axillary pain | 4 | 3 | 16 |
| Blood pressure increased | 4 | 3 | 4 |
| Dyspnoea | 4 | 2 | 6 |
| Menstruation delayed | 4 | 4 | 3 |
| Muscle spasms | 4 | 4 | 3 |
| Parosmia | 4 | 4 | 1 |
| Pruritus | 4 | 3 | 5 |
| Rash | 4 | 3 | 5 |
| Somnolence | 4 | 3 | - |
| Chest pain | 3 | 2 | 5 |
| Condition aggravated | 3 | 1 | - |
| Disturbance in attention | 3 | 2 | 2 |
| Dysgeusia | 3 | 3 | - |
| Feeling abnormal | 3 | 2 | 4 |
| Feeling cold | 3 | 2 | - |
| Hot flush | 3 | 3 | 6 |
| Hypoaesthesia | 3 | - | 2 |
| Limb discomfort | 3 | 2 | 2 |
| Memory impairment | 3 | 2 | 1 |
| Ocular discomfort | 3 | 1 | 1 |
| Oral herpes | 3 | 1 | 2 |
| SARS-CoV-2 test positive | 3 | - | 1 |
| Vomiting | 3 | 3 | 8 |
| Aphthous ulcer | 2 | 2 | - |
| Blood pressure decreased | 2 | - | 1 |
| Burning sensation | 2 | 2 | 1 |
| Dizziness | 2 | 2 | 8 |
| Dysentery | 2 | - | - |
| Dysphonia | 2 | - | 1 |
| Eye irritation | 2 | 2 | 1 |
| Haematochezia | 2 | 2 | - |
| Heart rate increased | 2 | - | 1 |
| Heavy menstrual bleeding | 2 | - | 1 |
| Hypomenorrhoea | 2 | 2 | - |
| Hypotension | 2 | 2 | - |
| Injected limb mobility decreased | 2 | 1 | 3 |
| Injection site hypoaesthesia | 2 | 1 | - |
| Injection site paraesthesia | 2 | 1 | 1 |
| Lip oedema | 2 | 1 | - |
| Muscular weakness | 2 | 1 | - |
| Neck pain | 2 | 1 | 2 |
| Oropharyngeal discomfort | 2 | 2 | - |
| Polymenorrhoea | 2 | 2 | - |
| Rash macular | 2 | 2 | 1 |
| Swollen tongue | 2 | 2 | - |
| Taste disorder | 2 | 1 | 1 |
| Abdominal discomfort | 1 | - | 1 |
| Abdominal distension | 1 | 1 | 1 |
| Abdominal pain upper | 1 | 1 | 3 |
| Anxiety | 1 | 1 | - |
| Aquagenic wrinkling of palms | 1 | 1 | - |
| Asthma | 1 | - | 1 |
| Auditory disorder | 1 | - | - |
| Back pain | 1 | 1 | 5 |
| Blood immunoglobulin M increased | 1 | 1 | - |
| Blood pressure systolic increased | 1 | 1 | - |
| Breast inflammation | 1 | 1 | - |
| Capillary disorder | 1 | - | - |
| Chest discomfort | 1 | 1 | 4 |
| Choking sensation | 1 | 1 | - |
| Confusional state | 1 | 1 | 4 |
| Constipation | 1 | 1 | - |
| Contusion | 1 | - | 1 |
| Dehydration | 1 | 1 | - |
| Dermatitis allergic | 1 | 1 | 1 |
| Diplopia | 1 | 1 | - |
| Dry throat | 1 | 1 | - |
| Dysmenorrhoea | 1 | 1 | - |
| Dysphagia | 1 | 1 | - |
| Dysstasia | 1 | 1 | - |
| Ear discomfort | 1 | 1 | 2 |
| Epicondylitis | 1 | 1 | - |
| Epistaxis | 1 | 1 | - |
| Erythema | 1 | - | 1 |
| Euphoric mood | 1 | 1 | - |
| Exertional headache | 1 | 1 | - |
| Eye pain | 1 | 1 | 2 |
| Fall | 1 | 1 | - |
| Flushing | 1 | - | - |
| Folliculitis | 1 | 1 | - |
| Gastrointestinal disorder | 1 | 1 | - |
| Haemorrhoids thrombosed | 1 | 1 | - |
| Head discomfort | 1 | 1 | 1 |
| Hepatitis | 1 | 1 | - |
| Herpes simplex | 1 | 1 | - |
| Herpes zoster | 1 | 1 | - |
| Hypoaesthesia oral | 1 | 1 | - |
| Increased appetite | 1 | - | - |
| Inflammation | 1 | 1 | - |
| Initial insomnia | 1 | 1 | - |
| Injection site urticaria | 1 | 1 | 1 |
| Insomnia | 1 | 1 | 3 |
| Intermenstrual bleeding | 1 | 1 | - |
| Joint swelling | 1 | - | - |
| Language disorder | 1 | - | - |
| Ligament injury | 1 | 1 | - |
| Light chain analysis increased | 1 | 1 | - |
| Lip swelling | 1 | 1 | - |
| Menstrual disorder | 1 | 1 | 6 |
| Muscle contractions involuntary | 1 | 1 | - |
| Myoclonus | 1 | 1 | - |
| Nasal discomfort | 1 | - | - |
| Ocular hyperaemia | 1 | 1 | - |
| Oedema | 1 | 1 | - |
| Oral mucosal blistering | 1 | 1 | - |
| Pain | 1 | 1 | 1 |
| Pancreatic enzymes increased | 1 | 1 | - |
| Pelvic pain | 1 | 1 | - |
| Periorbital swelling | 1 | - | - |
| Peripheral coldness | 1 | 1 | 1 |
| Pharyngeal oedema | 1 | 1 | - |
| Presyncope | 1 | 1 | 2 |
| Proteinuria | 1 | - | - |
| Psoriatic arthropathy | 1 | 1 | - |
| Psychomotor hyperactivity | 1 | 1 | - |
| Rash pruritic | 1 | 1 | - |
| Red blood cell count decreased | 1 | 1 | - |
| Restlessness | 1 | - | 1 |
| Rhinitis allergic | 1 | 1 | - |
| Rhinorrhoea | 1 | - | - |
| Rotator cuff syndrome | 1 | 1 | - |
| Salivary hypersecretion | 1 | - | - |
| Sciatica | 1 | 1 | 2 |
| Skin discolouration | 1 | 1 | - |
| Skin odour abnormal | 1 | 1 | - |
| Sneezing | 1 | 1 | - |
| Swelling | 1 | 1 | 2 |
| Swelling face | 1 | - | - |
| Temperature intolerance | 1 | - | - |
| Tenosynovitis | 1 | 1 | - |
| Tension headache | 1 | - | 1 |
| Throat irritation | 1 | 1 | - |
| Thrombophlebitis | 1 | 1 | - |
| Tongue pruritus | 1 | - | - |
| Tonsillar hypertrophy | 1 | 1 | - |
| Trigger finger | 1 | - | - |
| Upper respiratory tract irritation | 1 | - | - |
| Palpitations | - | 2 | - |
| COVID-19 | - | - | 4 |
| Dyspepsia | - | - | 4 |
| Etrasystoles | - | - | 3 |
| Hyperhidrosis | - | - | 3 |
| Injection sute rash | - | - | 3 |
| Arrhythmia | - | - | 2 |
| Body temperature decreased | - | - | 2 |
| Bronchitis | - | - | 2 |
| Dermatitis | - | - | 2 |
| Influenza like illness | - | - | 2 |
| Night sweats | - | - | 2 |
| Oedema peripheral | - | - | 2 |
| Paraesthesia oral | - | - | 2 |
| Sleep disorder | - | - | 2 |
| Somnolence | - | - | 2 |
| Visual impairment | - | - | 2 |
| Ageusia | - | - | 1 |
| Allodynia | - | - | 1 |
| Anosmia | - | - | 1 |
| Asthenia | - | - | 1 |
| Atrial tachycardia | - | - | 1 |
| Bone pain | - | - | 1 |
| Cardiomyopathy | - | - | 1 |
| Cold sweat | - | - | 1 |
| Cough variant asthma | - | - | 1 |
| Cystitis | - | - | 1 |
| Depressed mood | - | - | 1 |
| Depression | - | - | 1 |
| Dermatitis bullous | - | - | 1 |
| Discomfort | - | - | 1 |
| Dry skin | - | - | 1 |
| Disorientation | - | - | 1 |
| Dyspnoea exertional | - | - | 1 |
| Eczema | - | - | 1 |
| Eye discharge | - | - | 1 |
| Eyelid myokymia | - | - | 1 |
| Facial pain | - | - | 1 |
| Fibromyalgia | - | - | 1 |
| Gastric cancer | - | - | 1 |
| Gastritis | - | - | 1 |
| Gastrooesophageal reflux disease | - | - | 1 |
| Genital pain | - | - | 1 |
| Genital rash | - | - | 1 |
| Genital swelling | - | - | 1 |
| Gingival bleeding | - | - | 1 |
| Goitre | - | - | 1 |
| Haemangioma of skin | - | - | 1 |
| Hepatic pain | - | - | 1 |
| Hordeolum | - | - | 1 |
| Hyperaesthesia teeth | - | - | 1 |
| Hyperglycaemia | - | - | 1 |
| Hypertension | - | - | 1 |
| Hypertensive crisis | - | - | 1 |
| Hypoacusis | - | - | 1 |
| Hyporeflexia | - | - | 1 |
| Infarction | - | - | 1 |
| Injection site joint inflammation | - | - | 1 |
| Lacrimation increased | - | - | 1 |
| Loss of consciousness | - | - | 1 |
| Lymph node pain | - | - | 1 |
| Mental fatigue | - | - | 1 |
| Micturition urgency | - | - | 1 |
| Musculoskeletal chest pain | - | - | 1 |
| Musculoskeletal pain | - | - | 1 |
| Musculoskeletal stiffness | - | - | 1 |
| Odynophagia | - | - | 1 |
| Oligomenorrhoea | - | - | 1 |
| Oxygen saturation decreased | - | - | 1 |
| Peripheral swelling | - | - | 1 |
| Pharyngitis | - | - | 1 |
| Photophobia | - | - | 1 |
| Pneumothorax | - | - | 1 |
| Psoriasis | - | - | 1 |
| Rheumatoid arthritis | - | - | 1 |
| Rhinitis | - | - | 1 |
| Sinusitis | - | - | 1 |
| Skin irritation | - | - | 1 |
| Syncope | - | - | 1 |
| Tendonitis | - | - | 1 |
| Thirst | - | - | 1 |
| Tongue discomfort | - | - | 1 |
| Toothache | - | - | 1 |
| Tremor | - | - | 1 |
| Vaccination site joint swelling | - | - | 1 |
| Vaginal haemorrhage | - | - | 1 |
| Varicose vein | - | - | 1 |
| Vascular pain | - | - | 1 |
| Vitiligo | - | - | 1 |
| Vitreous floaters | - | - | 1 |

**Supplementary table 4.** List of the overall reported serious ADRs (MedDRA Preferred Terms), following a first, second or booster dose of any COVID-19 vaccine.

|  | First dose | Second dose | Booster dose |
| --- | --- | --- | --- |
| Arthralgia | 1 | - | - |
| Body temperature increased | 1 | 1 | - |
| Condition aggravated | 1 | - | - |
| Dysentery | 1 | 1 | - |
| Haematochezia | 1 | 1 | - |
| Headache | 1 | - | - |
| Herpes zoster | 1 | 1 | - |
| Hyperpyrexia | 1 | - | 2 |
| Malaise | 1 | - | - |
| Myalgia | 1 | - | - |
| Paraesthesia | 1 | - | - |
| Pyrexia | 1 | - | 4 |
| Tenosynovitis | 1 | 1 | - |
| Urticaria | 1 | - | - |
| Arrhythmia | - | - | 1 |
| Cystitis | - | - | 1 |
| Gastritis | - | - | 1 |
| Infarction | - | - | 1 |
| Loss of consciousness | - | - | 1 |
| Pneumothorax | - | - | 1 |

**Annex I.** Web-based questionnaires and response items (also available in the study protocol).

## Baseline questionnaire (compulsory)

- Gender
- Age (e.g. calculated based on date of birth)
- National identification number, if the data can be linked to a vaccination register
- Geographical area (e.g local health unit)
- Planned vaccination date
- Medical history (current conditions) and pregnancy (closed questions):
  - Impaired immune function (e.g. due to disease or due to treatment)
  - Lung disease (including chronic obstructive pulmonary disease and asthma)
  - Liver disease
  - Neurological disease or injury (including epilepsy)
  - Psychiatric condition (including depression)
  - Cardiovascular disease
  - Hypertension
  - Kidney disease
  - Diabetes
  - Malignancy / cancer
  - Allergy (y/n) with subquestion (checkbox): pollen (hay fever), dust mites, animal (e.g. cat), food (e.g. egg), insect bites and stings, medication, other (namely:…)
  - Other disease:…
  - Pregnancy (subquestion on gestational age)
  - None of the above
- Health care worker/ informal caregiver (y/n)? If yes sub question (radio button): medical doctor, pharmacist, nurse, other paramedical (e.g. midwife, physiotherapist), informal caregiver, other (namely:…)
- Previous infection with SARS-CoV-2 / COVID-19 disease (yes, confirmed with a test; yes but no test; probably but no test; no)?
  - Date of symptom onset
  - Severity (asymptomatic, cold-like symptoms, considerable symptoms without hospitalisation, hospitalized due to symptoms)
- Height (in cm)
- Weight (in kg)
- Current medication (including over the counter medication; ATC-coded locally)
- Vaccinations (other than COVID-19 vaccine) in the past 2 years (namely: …)

## Extra questions for identification of special groups

- Have you ever experienced an allergic reaction in the past after receiving a vaccine (of any kind)?
- Have you ever had any allergic reaction (e.g. anaphylactic shock) that has required emergency treatment or A&E admission?
- Did you take any medication (e.g., antihistamines or corticosteroids) before COVID-19 vaccination to prevent vaccine-related allergy?
  - Please specify:_______
- Are you immunocompromised due to any medical conditions (e.g., HIV/AIDS, transplants, autoimmune diseases, leukaemia/lymphoma)?
  - HIV/AIDS
  - Transplantation
  - Leukaemia/lymphoma
  - Autoimmune diseases
  - Other, please specify:_______
- Do you currently take any medications that affect your immune system (e.g., chemotherapy, glucocorticoids, anti-rheumatics)?
  - No
  - Yes, Please specify:___________
- Will or have you stopped your medication in the period immediately preceding or following the COVID-19 vaccination?
  - No
  - Yes, I have stopped/will stop my medication preceding my COVID-19 vaccination
  - Yes, I have stopped/will stop my medication following my COVID-19 vaccination
  - Yes, I have stopped/will stop my medication both in the period preceding and following my COVID-19 vaccination

**Extra baseline questions for pregnant women**

- When is your baby due? (You can work this out by counting 40 weeks from the first day of your last period)
- Dd/mm/20yy (example: 15/March/2022)
- How many weeks pregnant are you? ______ weeks
- Are you pregnant with more than one baby? (Yes / No / Not sure or don’t know yet / Prefer not to say)
- During this pregnancy or before this pregnancy, have you experienced or been treated for any of the following conditions? (Please tick Yes or No for each condition)
  - Diabetes (Yes, during this pregnancy/ Yes, before this pregnancy / No)
  - High blood pressure (hypertension) (Yes, during this pregnancy / Yes, before this pregnancy / No)
  - Blood clots (thrombosis) (Yes, during this pregnancy / Yes, before this pregnancy / No)
  - Obesity (Yes, during this pregnancy / Yes, before this pregnancy / No)
- Before this pregnancy, how many times have you been pregnant? ______ times (force the 0 to 10)
- Please indicate the number of babies born full term (after 39 weeks). ______ babies born full term
- Please indicate the number of babies born preterm (born alive before 39 weeks of pregnancy)

______ babies born preterm

Have you ever had a caesarean section (this is when the baby is removed by an incision-cut in the mum’s belly)? (yes, no, prefer not to say, not sure)

- Please, specify how many times you had a caesarean section. _______ times
- Have you ever experienced a stillbirth before (loss of your baby after 20 weeks of pregnancy)? (yes, no, prefer not to say, not sure)
- Please, specify how many times you experienced a stillbirth. _________ times
- Have you ever experienced a miscarriage? (yes, no, prefer not to say, not sure)
- Please, specify how many times you experienced a miscarriage. _________ times
- Have you ever had an ectopic pregnancy (pregnancy growing outside of your uterus)? (yes, no, prefer not to say, not sure)

## Additional component to baseline questionnaire – if already vaccinated

- COVID-19 vaccination date
- Immunizer (e.g. GP, employer, municipal health authority, etc.)
- Vaccination site (arm which one…)
- Antipyretics intake (if applicable, as prophylaxis)
- Vaccine brand (GTIN code) and batch number: It should be ensured that the vaccine recipients receive this information themselves, e.g. through a vaccination certificate that is obtained at the point of vaccination, in a vaccination booklet that is updated at the point of vaccination, and/or - less preferable - that they can look it up in a digital account (e.g. linked with the vaccination register). The participant can then either report the name of vaccine brand or the GTIN, or upload a photo (e.g. of the barcode or GTIN) to the LIM web app.
  - In addition, this information should be derived from a vaccination register to improve data quality / completeness.

## Verification of vaccination on planned vaccination date (dose 1)

- Have you received the vaccination?
  - If yes:
    - COVID-19 vaccination date
    - Immunizer (e.g. GP, employer, municipal health authority, etc.)
    - Antipyretics intake (if applicable, as prophylaxis)
    - Vaccine brand (GTIN code) and batch number: It should be ensured that the vaccine recipients receive this information themselves, e.g. through a vaccination certificate that is obtained at the point of vaccination, in a vaccination booklet that is updated at the point of vaccination, and/or - less preferable - that they can look it up in a digital account (e.g. linked with the vaccination register). The participant can then either report the name of vaccine brand or the GTIN, or upload a photo (e.g. of the barcode or GTIN) to the LIM web app.
      - In addition, this information should be derived from a vaccination register to improve data quality / completeness.
  - If no:
    - New planned date
      - This same questionnaire will be sent on the new planned date

## Q1: 7 days after dose 1

- Have you experienced an adverse reaction vaccination (y/n)? If yes:
- Injection site reaction on the right side (closed question)
  - - Sub-question (closed) on symptoms (redness, warmth, pain, itch, haematoma, swelling, induration)
      - Closed subquestion to assess extensive limb swelling (if swelling and/or redness are ticked)
- Injection site reaction on the left side (closed question)
  - - Subquestion (closed) on symptoms (redness, warmth, pain, itch, haematoma, swelling, induration)
      - Closed subquestion to assess extensive limb swelling (if swelling and/or redness are ticked)
- Fever (closed question) - sub question on highest temperature that was measured:
  - - Category:
      - 37.5 – 37.9 degrees Celsius
      - 38.0 – 40.4 degrees Celsius
      - 40.5 – 42.0 degrees Celsius
      - Higher than 42 degrees Celsius
      - Not measured
    - Temperature as continuous variable (1 decimal)
- Chills (closed question),
- Headache (closed question),
- Nausea (closed question),
- Myalgia / muscle pain (closed question),
- Arthralgia / joint pain (closed question),
- Malaise (closed question),
- Fatigue (closed question),
  - Other ADR (open question)
- Information collected for each reported ADR:
  - Latency (i.e. date of onset as well as in seconds, minutes, hours, days after vaccination)
  - Outcome (recovered, recovering, not recovered)
    - If recovered: duration of symptoms (date as well as in seconds, minutes, hours, days after onset)
  - Visited a medical doctor/GP because of the adverse reaction? (if there were tests done, the outcomes of these tests will be asked, e.g. blood test or ECG)
  - Was the adverse reaction treated? (including over the counter medication; ATC-coded locally)
  - Impact of the reaction (5-point scale from not severe to very severe)
  - Seriousness according to CIOMS (hospitalisation >24h; life-threatening situation; other medically important reaction). If ticked: open sub-questions.
  - Possibility to upload a picture of the reaction and/or documents such as a hospital discharge letter (participant should not be identifiable).

## Q2: 3 weeks after dose 1

Old adverse reactions:

- Outcome of each of the ADRs from which the participant had not (yet) recovered in the previous questionnaire (recovered, recovering, not recovered)
  - If recovered: duration of symptoms (date as well as in seconds, minutes, hours, days, weeks after onset)
- Visited a medical doctor/GP because of the adverse reaction? (if there were tests done, the outcomes of these tests will be asked, e.g. blood test or ECG)
- Was the adverse reaction treated? (including over the counter medication; ATC-coded locally)
- Impact of the adverse reaction (5-point scale from not severe to very severe)
- Seriousness according to CIOMS (hospitalisation >24h; life-threatening situation; other medically important adverse reaction). If ticked: open sub-questions.
- Possibility to upload a picture of the adverse reaction and/or documents such as a hospital discharge letter (participant should not be identifiable).

New adverse reactions: Identical to Q1

### Q3 & Q4: 5 & 8 weeks after dose 1

Identical to Q2, and in addition, it includes the following questions:

- Have you received a second dose of the vaccination?
  - If yes:
    - COVID-19 vaccination date
    - Immunizer (e.g. GP, employer, municipal health authority, etc.)
    - Antipyretics intake (if applicable, as prophylaxis)
    - Vaccine brand (GTIN code) and batch number: It should be ensured that the vaccine recipients receive this information themselves, e.g. through a vaccination certificate that is obtained at the point of vaccination, in a vaccination booklet that is updated at the point of vaccination, and/or - less preferable - that they can look it up in a digital account (e.g. linked with the vaccination register). The participant can then either report the name of vaccine brand or the GTIN, or upload a photo (e.g. of the barcode or GTIN) to the LIM web app.
      - In addition, this information should be derived from a vaccination register to improve data quality / completeness.
  - If not: reason for not taking it or for delay? (practical reason, because of the experienced side effects of the first dose, other)

### Q5: 3 months after dose 1

Identical to Q3 & Q4, and in addition, it includes the following questions:

- Infection with SARS-CoV2 / COVID-19 disease since vaccination? (yes, confirmed with a test; yes but no test; probably but no test; no)?
  - Date of symptom onset
  - Severity (asymptomatic, cold-like symptoms, considerable symptoms without hospitalisation, hospitalized due to symptoms)

### Q6: 6 months after dose 1

Identical to Q5, except that 2 questions are adapted as follows:

- Infection with SARS-CoV2 / COVID-19 disease since the last questionnaire? (yes, confirmed with a test; yes but no test; probably but no test; no)?
  - Date of symptom onset
  - Severity (asymptomatic, cold-like symptoms, considerable symptoms without hospitalisation, hospitalized due to symptoms)

### “End of Pregnancy” questionnaire: 45 days after due date

**Your pregnancy and delivery (Pregnancy outcome)**

- - - - Did you delivered your baby? (Yes / No, I experienced a stillbirth (loss of your baby after 20 weeks of pregnancy / No, I had a miscarriage (loss of your baby before 20 weeks of pregnancy) / No, please specify)
- How many weeks pregnant were you when you had your baby? (the time from the first day of your last menstrual period)? ________ weeks
- Have you been diagnosed with any of the following conditions during your pregnancy?
- Gestational diabetes (yes, no)
- High blood pressure (hypertension) (yes, no)
- Blood clots (thrombosis) (yes, no)
- Obesity (yes, no)
- Preeclampsia (yes, no)
- Intra uterine growth restriction (yes, no)
- Abnormal foetal doppler (yes, no)
- Threatened preterm labor (yes, no)
- Placenta praevia (yes, no)
- Premature Rupture of Membranes (yes, no)
- Placental abruption (yes, no)
- Other, please specifiy (yes, no)

### About your baby (Neonatal outcome)

- What is your baby’s gender? (Male/Female)
- Please, tell us your baby weight at birth if known (___Kg, or ___ pounds)
- And her/his height at birth if known (___cm, ___feet___inches)
- Has your baby died in his first two weeks of life? (yes, no, do not)
- Has your baby experienced any of the following conditions? Please tick all that apply
- neonatal intensive care unit admission for any reason
- physical birth defect (missing or malformed part of the body)
- infection (such as infection of the lungs, eye infection, diarrhoea, white/yellow patches in the mouth)
- hypoglycaemia (the level of sugar in the blood is too low)
- Physical injury at birth, that is the result of being born
- Breathing problems at birth (baby was breathing too fast or too slowly at birth)
- feeding problems at birth (baby had difficulty eating in the first two weeks of life)
- Hypothermia (difficulty to keep its body temperature)
- Jaundice (the colour of the skin was/is yellow)
- Other conditions you would like us to know about _________

To fill in only if answered No to Q 1.1 in the “End of Pregnancy” questionnaire. You answered that you experienced a stillbirth. If you would like to share what happened please fill in the blank space
