# Supplementary material for: Cohort event monitoring of safety of COVID-19 vaccines: the Italian experience of the “ilmiovaccinoCOVID19 collaborating group”
Source: Front Drug Saf Regul. 2024 Aug 12;4:1363086. doi: 10.3389/fdsfr.2024.1363086 (PMC12445166; doi:10.3389/fdsfr.2024.1363086)
Supplement: Supplementary file 4 [file DataSheet3.PDF]

Il tuo contributo **rende i vaccini più sicuri**

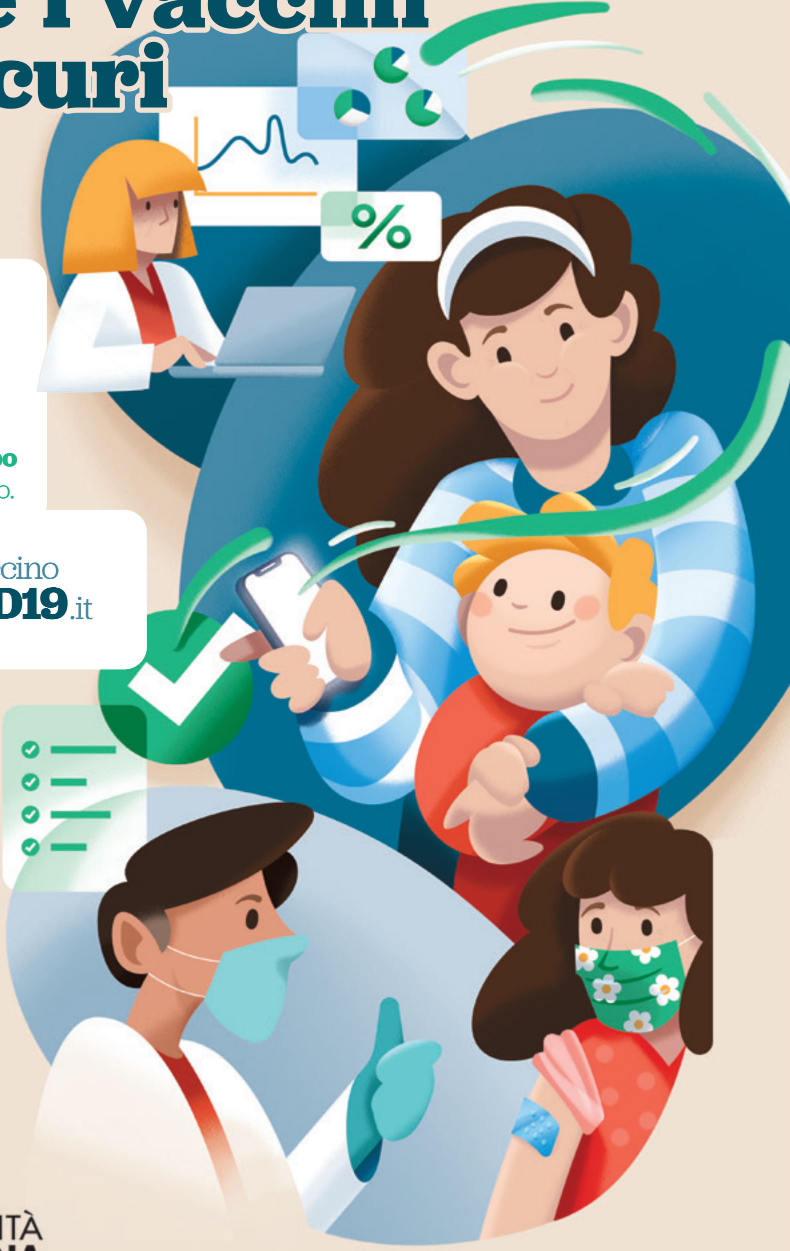

Partecipa al monitoraggio degli effetti collaterali dei vaccini anti COVID-19 registrandoti al sito web **fino a 48 ore dopo** aver ricevuto il vaccino.

ilmiovaccino **COVID19**.it

UNIVERSITÀ di **VERONA**

Dipartimento di **DIAGNOSTICA E SANITÀ PUBBLICA**

Hai avuto qualche effetto collaterale dopo il vaccino? Questa informazione è importante

Questo studio è stato finanziato dall'Agenzia Europea del Farmaco (EMA) che è responsabile dell'approvazione e del monitoraggio post-marketing dei vaccini contro il COVID-19, insieme all'Agenzia Italiana del Farmaco (AIFA). Il Centro di coordinamento dell'Università di Verona, insieme ad altri partner Italiani ed internazionali da 16 Paesi Europei, sta raccogliendo informazioni sui possibili effetti collaterali in seguito alla vaccinazione anti-COVID-19. Partecipa anche tu.

Rendere i vaccini più sicuri

I vaccini contro il COVID-19 sono stati studiati a fondo e soddisfano i requisiti di sicurezza. Tuttavia, possono ancora verificarsi effetti collaterali inattesi. Non si sa con quale frequenza possono verificarsi o se alcune persone hanno maggiori probabilità di sperimentarli. **Sarà in particolare condotto un monitoraggio degli effetti collaterali dei vaccini nelle seguenti categorie di persone: bambini/adolescenti, persone con storia di allergia, pazienti immunocompromessi (es. trapiantati, in trattamento con immunosoppressori, affetti da patologie autoimmuni), e persone con pregressa infezione da SARS-CoV-2.** Partecipando a questo studio puoi aiutarci a ottenere maggiori informazioni al riguardo e rendere l'uso dei vaccini ancora più sicuro. Tutte le informazioni raccolte saranno condivise con l'EMA e l'AIFA.

Partecipare è semplice

Stai per ricevere o hai ricevuto da non più di 48 ore il vaccino contro il COVID-19?
Partecipa allo studio registrandoti al seguente sito web: **www.ilmiovaccinocovid19.it**

Sul sito web troverai inoltre ulteriori informazioni sullo studio. Dopo la registrazione riceverai via e-mail 1 questionario di base e 6 questionari di follow-up nei sei mesi successivi alla ricezione della prima dose di vaccino. Ti verranno poste domande sulla tua salute e sui possibili effetti collaterali. La compilazione del questionario richiederà circa 10 minuti.
È possibile partecipare solo ed esclusivamente fino a 48 ore dopo la vaccinazione.
Puoi decidere di interrompere la partecipazione allo studio in qualsiasi momento.

Partecipa e vai su
www.ilmiovaccinoCOVID19.it

Questo studio è coordinato dall'Università degli Studi di Verona. I tuoi dati personali saranno trattati con riservatezza.

CON LA PARTECIPAZIONE DI
