# Supplementary material for: Cohort event monitoring of safety of COVID-19 vaccines: the Italian experience of the “ilmiovaccinoCOVID19 collaborating group”
Source: Front Drug Saf Regul. 2024 Aug 12;4:1363086. doi: 10.3389/fdsfr.2024.1363086 (PMC12445166; doi:10.3389/fdsfr.2024.1363086)
Supplement: Supplementary file 5 [file DataSheet1.PDF]

# Il tuo contributo **rende i vaccini più sicuri**

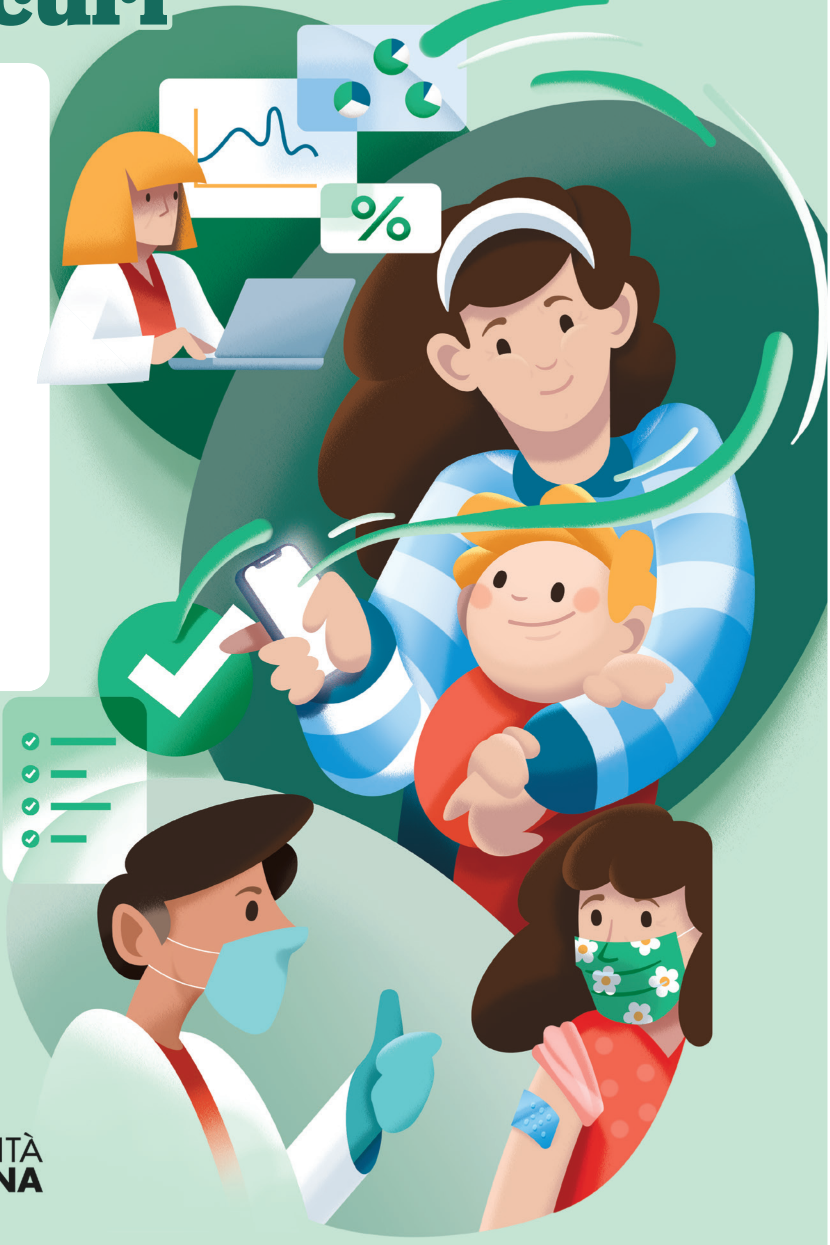


Partecipa al monitoraggio degli effetti collaterali dei vaccini anti COVID-19 registrandoti al sito web **fino a 48 ore dopo** aver ricevuto la **dose di richiamo o la terza dose** di vaccino.

**covidvaccine**
monitor.eu/it


UNIVERSITÀ di VERONA
Dipartimento di DIAGNOSTICA E SANITÀ PUBBLICA

## **Hai ricevuto la terza dose o la dose di richiamo del vaccino contro il COVID-19?** Puoi segnalarci qualunque effetto collaterale, è importante!

Questo studio è stato finanziato dall'Agenzia Europea del Farmaco (EMA), responsabile insieme all'Agenzia Italiana del Farmaco (AIFA) dell'approvazione e del monitoraggio dei vaccini contro il COVID-19. Il Centro di coordinamento dell'Università di Verona, insieme ad altri partner italiani ed internazionali (16 Paesi EU e non EU), sta raccogliendo informazioni sui possibili effetti collaterali in seguito alla vaccinazione contro il COVID-19. Partecipa anche tu!

## Rendere i vaccini **più sicuri**

I vaccini contro il COVID-19 sono stati studiati a fondo e soddisfano tutti i requisiti di sicurezza previsti per qualsiasi altro vaccino. Tuttavia, potrebbero ancora verificarsi effetti collaterali inattesi. Non si sa con quale frequenza questi potrebbero verificarsi o se alcune persone hanno maggiori probabilità di sperimentarli. Sarà in particolare condotto un monitoraggio della risposta alla vaccinazione nei **vaccinati che ricevono la terza dose o la dose di richiamo** che hanno come obiettivo quello di migliorare il livello di protezione contro il COVID-19. Partecipando a questo studio puoi aiutarci a raccogliere importanti informazioni per rendere l'uso dei vaccini ancora più sicuro. Tutte le informazioni raccolte saranno condivise con i sistemi di farmacovigilanza di EMA e AIFA e confrontate con quelle degli altri paesi.

## Partecipare **è semplice**

**Hai ricevuto la terza dose o la dose di richiamo** del vaccino contro il COVID-19 **da meno di 48 ore?** La dose di richiamo contro il COVID-19 viene somministrata dopo aver completato un ciclo vaccinale, normalmente dopo 6 mesi dall'ultima somministrazione di vaccino contro il COVID-19. In caso di severa compromissione del sistema immunitario, la terza dose può essere invece somministrata già a partire da 28 giorni dall'ultima somministrazione di vaccino contro il COVID-19.

Partecipa allo studio registrandoti al sito web: **covidvaccinemonitor.eu/it.**

Sul sito troverai tutte le informazioni necessarie per partecipare. Dopo la registrazione riceverai una e-mail con le indicazioni per compilare un questionario di base. Riceverai cinque questionari di follow-up nei tre mesi successivi.

## Cosa **vogliamo sapere**

Nei questionari troverai domande sulla tua salute e sui possibili effetti collaterali che potrebbero essere insorti dopo la vaccinazione. La compilazione di ogni questionario non impegnerà più di 10 minuti; ricorda che è possibile partecipare **fino a 48 ore** dopo aver ricevuto la **terza dose o la dose di richiamo del vaccino.** Se dovessi cambiare idea, nessun problema: puoi decidere di ritirarti ed interrompere la partecipazione allo studio in qualsiasi momento, senza bisogno di fornire nessuna giustificazione e senza nessuna conseguenza.

**Partecipa allo studio, vai su**

**www.covidvaccinemonitor.eu/it**

**e contribuisci con noi alla sicurezza dei vaccini contro il COVID-19!**

Questo studio è coordinato dall'Università degli Studi di Verona.
I tuoi dati personali saranno trattati con riservatezza.

I loghi dei partner italiani partecipanti al progetto possono essere trovati sul sito: **covidvaccinemonitor.eu/it**
